# Novel chemotype NLRP3 inhibitors that target the CRID3-binding pocket with high potency

**DOI:** 10.26508/lsa.202402644

**Published:** 2024-03-22

**Authors:** Lieselotte Vande Walle, Madhukar Said, Oonagh Paerewijck, Arinna Bertoni, Marco Gattorno, Bruno Linclau, Mohamed Lamkanfi

**Affiliations:** 1 Laboratory of Medical Immunology, Department of Internal Medicine and Paediatrics, Ghent University , Ghent, Belgium; 2 Research Group Organic and Medicinal Chemistry, Department of Organic and Macromolecular Chemistry, Ghent University , Ghent, Belgium; 3 UOC Reumatologia e Malattie Autoinfiammatorie, IRCCS Istituto G. Gaslini, Genova, Italy

## Abstract

The NLRP3 inflammasome plays a central role in various human diseases. Despite significant interest, most clinical-grade NLRP3 inhibitors are derived from sulfonylurea inhibitor CRID3 (also called MCC950). Here, we describe a novel chemical class of NLRP3-inhibiting compounds (NIC) that exhibit potent and selective NLRP3 inflammasome inhibition in human monocytes and mouse macrophages. BRET assays demonstrate that they physically interact with NLRP3. Structural modeling further reveals they occupy the same binding site of CRID3 but in a critically different conformation. Furthermore, we show that NIC-11 and NIC-12 lack the off-target activity of CRID3 against the enzymatic activity of carbonic anhydrases I and II. NIC-12 selectively reduces circulating IL-1ß levels in the LPS-endotoxemia model in mice and inhibits NLRP3 inflammasome activation in CAPS patient monocytes and mouse macrophages with about tenfold increased potency compared with CRID3. Altogether, this study unveils a new chemical class of highly potent and selective NLRP3-targeted inhibitors with a well-defined molecular mechanism to complement existing CRID3-based NLRP3 inhibitors in pharmacological studies and serve as novel chemical leads for the development of NLRP3-targeted therapies.

## Abstract (precis)

The NLRP3 inflammasome drives various diseases. Here, we describe novel potent and selective NLRP3-targeted inhibitors to bolster pharmacological studies and development of NLRP3-targeted therapies.

## Introduction

Inflammasomes play a central role in orchestrating innate and adaptive immune responses. Assembly of these intracellular multi-protein complexes results in recruitment and activation of caspase-1. This protease is a prototypic and evolutionarily conserved cysteine-dependent endoprotease and assumes a critical function in the maturation of pro-interleukin (IL)-1β and pro-IL-18, converting them into their mature and active forms, IL-1β and IL-18, respectively (Van Opdenbosch & Lamkanfi, 2019). Furthermore, caspase-1 plays a pivotal role in the cleavage of gasdermin D (GSDMD), a cytosolic protein that instigates the formation of large GSDMD pores, thereby disrupting ionic gradients across cellular membranes. This process, known as pyroptosis, entails a highly inflammatory lytic cell death mechanism characterized by plasma membrane rupture, facilitating the extracellular dispersion of cytokines, damage-associated molecular patterns such as high mobility group box 1, and other cytosolic solutes such as the lytic cell death marker lactate dehydrogenase (LDH).

The NLRP3 inflammasome has garnered substantial interest as a therapeutic target because of its pathogenic role across a spectrum of human diseases. This includes conditions such as gout and pseudogout, metabolic disorders like atherosclerosis and nonalcoholic fatty liver disease/nonalcoholic steatohepatitis (NAFLD/NASH), and neurodegenerative diseases like Alzheimer’s disease, Parkinson’s disease, and multiple sclerosis (Mangan et al, 2018). Furthermore, gain-of-function mutations occurring within or in the close vicinity of the central NACHT domain of NLRP3 give rise to three autosomal dominantly inherited periodic fever syndromes collectively referred to as cryopyrin-associated periodic syndrome (CAPS). The clinical manifestations of CAPS exhibit a spectrum of severity, with familial cold autoinflammatory syndrome representing the mildest form, Muckle–Wells syndrome (MWS) demonstrating moderate severity, and neonatal-onset multisystem inflammatory disease/chronic infantile neurological, cutaneous and articular syndrome manifesting as the most severe subtype of CAPS (Van Opdenbosch & Lamkanfi, 2019). Because of its extensive involvement across disease indications, selective and potent inhibitors of the NLRP3 inflammasome have considerable therapeutic potential (Vande Walle & Lamkanfi, 2024). Early studies with sulfonylurea compounds served as proof of concept for achieving selective inhibition of the NLRP3 inflammasome using small molecules (Lamkanfi et al, 2009; Coll et al, 2015). Among these, CRID3 (also named MCC950) stands out as an inhibitor that exhibits selective NLRP3 inhibition with nM potency, and it is widely used in preclinical studies (Mangan et al, 2018; Vande Walle & Lamkanfi, 2024). In addition, structural biology studies, utilizing both X-ray crystallography and cryo-electron microscopy, have recently elucidated the binding pocket of CRID3 at a cleft situated at the interface of the four subdomains constituting the central NACHT region of NLRP3 (Dekker et al, 2021; Hochheiser et al, 2022; McBride et al, 2022). This structural insight provides valuable knowledge regarding the mechanistic basis of NLRP3 inhibition by CRID3, revealing that CRID3 binding in this pocket maintains NLRP3 in an inactive conformation by preventing ADP/ATP exchange (Dekker et al, 2021; Hochheiser et al, 2022; McBride et al, 2022). Capitalizing on these scientific breakthroughs, several therapeutic NLRP3 inhibitors based on the CRID3 scaffold are under development, with the most advanced candidates currently undergoing phase 2 clinical trials in humans (Vande Walle & Lamkanfi, 2024).

However, the inhibitory potency of CRID3 is diminished by disease-associated mutations in and around the NLRP3 NACHT in preclinical mouse models of CAPS and in LPS-stimulated PBMCs of CAPS patients (Vande Walle et al, 2019; Weber et al, 2022; Vande Walle & Lamkanfi, 2023). Furthermore, carbonic anhydrase II (CA II) was recently identified as an off-target for this compound class (Kennedy et al, 2021). Another concern is that early clinical development of CRID3 in a phase 2 clinical trial for the treatment of rheumatoid arthritis was discontinued, reportedly because of CRID3 dosing in healthy volunteers causing drug-induced liver injury (Shah et al, 2015; Kennedy et al, 2021; Li et al, 2023). Therefore, the discovery of novel potent and selective NLRP3-targeted inhibitors with distinctive chemical scaffolds is urgently needed to open up additional avenues for clinical development of NLRP3-targeted therapeutics.

A screen in PubChem identified novel pyrolo-triazine acetamide compounds that inhibit nigericin-induced interleukin (IL)-1ß secretion from the immortalized human leukemia cell line THP-1 (Farady et al, 2020). We selected two representative compounds from this series, which we designated as NLRP3-inhibiting compounds (NIC)-11 and NIC-12, to functionally characterize their activity profile and mechanism of action against various NLRP3 inflammasome stimuli in primary immune cells of human and murine origin. We show that NIC-11 and NIC-12 selectively inhibited inflammasome activation by various NLRP3 stimuli with nM potency in both human blood monocytes and primary mouse BMDMs without targeting NF-κB–dependent priming or activation of the NLRC4, AIM2, and pyrin inflammasomes. Bioluminescence resonance energy transfer (BRET) analyses demonstrated that this novel class of inhibitors binds to the central NLRP3 NACHT domain in live cells. Subsequent structural modeling studies revealed that NIC-12 interacts with the recently discovered binding pocket of CRID3 in the central NLRP3 NACHT domain, but in a critically distinct conformation compared with CRID3. Unlike CRID3, NIC-11 and NIC-12 did not modulate the enzymatic activity of carbonic anhydrases I and II in biochemical assays. Finally, we demonstrate that NIC-12 suppresses circulating IL-1ß levels in vivo in LPS-challenged mice and inhibits NLRP3 inflammasome activation in primary monocytes from CAPS patients and macrophages expressing various disease-associated NLRP3 mutants with significantly increased potency compared with CRID3. In conclusion, the work presented here identifies a novel chemical class of NLRP3-targeted inhibitors and defines their binding pocket and molecular mechanism of action. These highly selective and active NLRP3 inhibitors may be used to complement existing CRID3-based NLRP3 inhibitors in pharmacological studies and serve as novel chemical leads for the development of NLRP3-targeted therapies.

## Results

### NIC-11 and NIC-12 inhibit NLRP3 inflammasome activation in mouse macrophages

**Figure 1.**
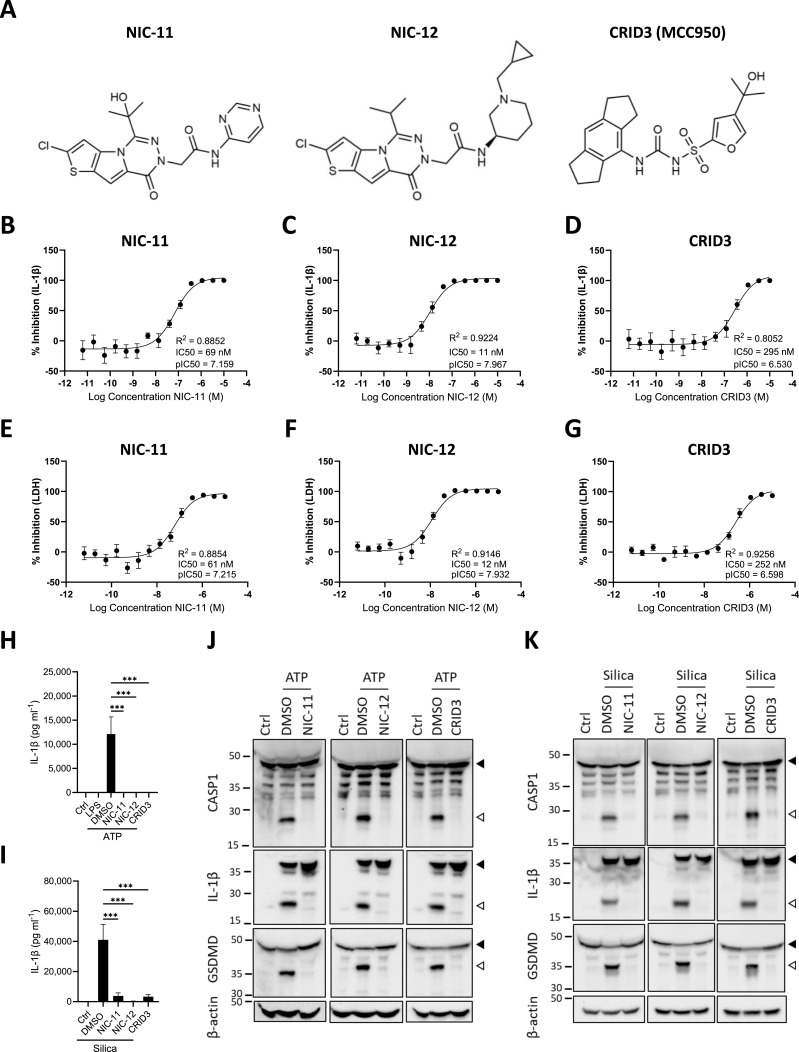
NIC-11 and NIC-12 inhibit NLRP3 inflammasome activation in mouse macrophages. **(A)** Chemical structures of NIC-11, NIC-12, and CRID3 (MCC950). **(B, C, D, E, F, G)** Dose–response curves of the effect of NIC-11 (B, E), NIC-12 (C, F), and CRID3 (D, G) on the secretion of IL-1β (B, C, D) or LDH (E, F, G) from LPS-primed (100 ng ml^−1^) primary BMDMs treated with nigericin (10 μM). IL-1β secretion and LDH release are depicted as mean ± SEM of n = 4 biological replicates. **(H, I, J, K)** Immortalized BMDM (iBMDM) were either left untreated (Ctrl) or primed with LPS (100 ng ml^−1^), followed by treatment with either 0.01% DMSO or 1 μM of the indicated compounds before being stimulated with ATP (5 mM) (H, J) or silica (500 ng ml^−1^) (I, K). **(H, I)** Supernatants were analyzed for IL-1β secretion. IL-1β secretion is depicted as mean ± SEM of n = 3 biological replicates. Statistical significance was analyzed by one-way ANOVA with Bonferroni’s multiple comparisons test. ***P ≤ 0.001. **(J, K)** Lysates were immunoblotted for caspase-1 (CASP1), IL-1β, GSDMD, and β-actin. One representative Western blot is shown out of three independent experiments. Arrows indicate the unprocessed (black) and processed (white) forms, respectively. Source data are available for this figure.

The defining tricyclic hexahydro-s-indacene moiety and central sulfonylurea group of the well-established CRID3 inhibitor class are absent from the chemical structures of NIC-11 and NIC-12 (Fig 1A). Instead, they carry a central pyrrole[1,2-d][1,2,4]triazine backbone that is extended with a chlorothiophene moiety at one end of the molecule. In NIC-11, an amide bond extends this thieno[2′,3′:4,5]pyrrole[1,2-d][1,2,4]triazine core with a pyrimidine sidechain. In NIC-12, the pyrimidine moiety is replaced with a substituted piperidine (Fig 1A).

**Figure S1.**
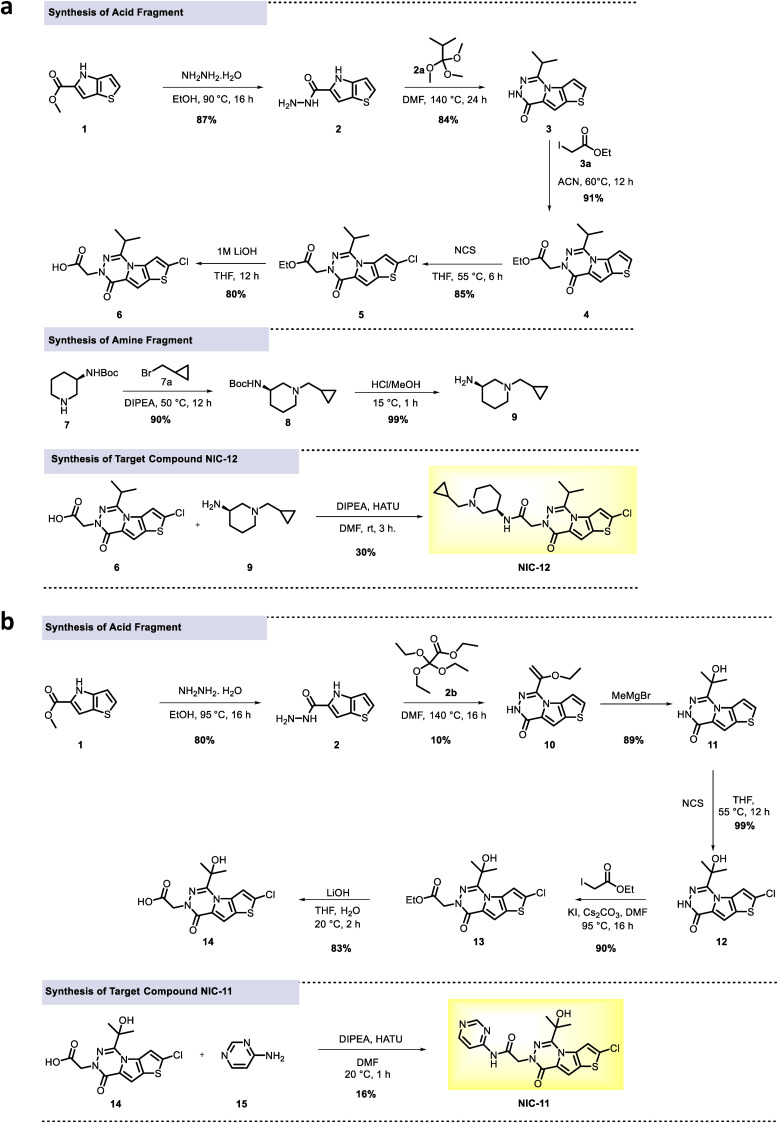
Synthesis schemes of NIC-11 and NIC-12. **(A, B)**, Target compounds NIC-12 (A) and NIC-11 (B) were synthesized from methyl 4H-thieno[3,2-b]pyrrole-5-carboxylate in, respectively, 8 and 7 steps by reacting the intermediate carbamate reagents with, respectively, (R)-1-(cyclopropylmethyl)piperidin-3-amine and 2-aminopyrimidine to generate NIC-12 and NIC-11, respectively.

After chemical synthesis (Fig S1) (Supplemental Data 1), we first evaluated the potential effects of NIC-11 and NIC-12 on the NLRP3 inflammasome by measuring their activity against nigericin-induced IL-1β release and pyroptosis induction in LPS-primed mouse BMDMs. CRID3 was taken along as a reference inhibitor in these analyses. NIC-11 and NIC-12 inhibited the release of IL-1β in a concentration-dependent manner with half-maximal inhibitory concentration (IC_50_) values of 69 nM and 11 nM, respectively (Fig 1B and C). The IC_50_ value of CRID3 in this assay was 295 nM (Fig 1D), indicating that NIC-11 and NIC-12 inhibit nigericin-induced IL-1β secretion from mouse BMDMs with apparent potencies that are, respectively, 4 and 26 times improved over that of the reference NLRP3 inhibitor CRID3. Consistent herewith, measurements of nigericin-induced pyroptosis showed that NIC-11 and NIC-12 dose-dependently inhibited LDH release with, respectively, 4 and 21 times higher potencies that CRID3 (Fig 1E–G).

Supplemental Data 1. Chemical synthesis.

The inhibitory activity of NIC-11 and NIC-12 was not confined to nigericin as a stimulus for NLRP3 inflammasome activation because IL-1ß secretion induced by ATP or silica crystals was equally blocked (Fig 1H and I). As seen with the reference inhibitor CRID3, immunoblotting analysis revealed that NIC-11 and NIC-12 act apically in the NLRP3 inflammasome pathway because NLRP3-induced caspase-1 maturation and caspase-1–mediated cleavage of proIL-1ß and the pyroptosis effector GSDMD were also inhibited (Fig 1J and K). Together, these results demonstrate that NIC-11 and NIC-12 represent a novel class of highly potent broad-spectrum NLRP3 inflammasome inhibitors.

### NIC-11 and NIC-12 target NLRP3 signaling upstream of caspase-1 in mouse BMDMs

**Figure 2.**
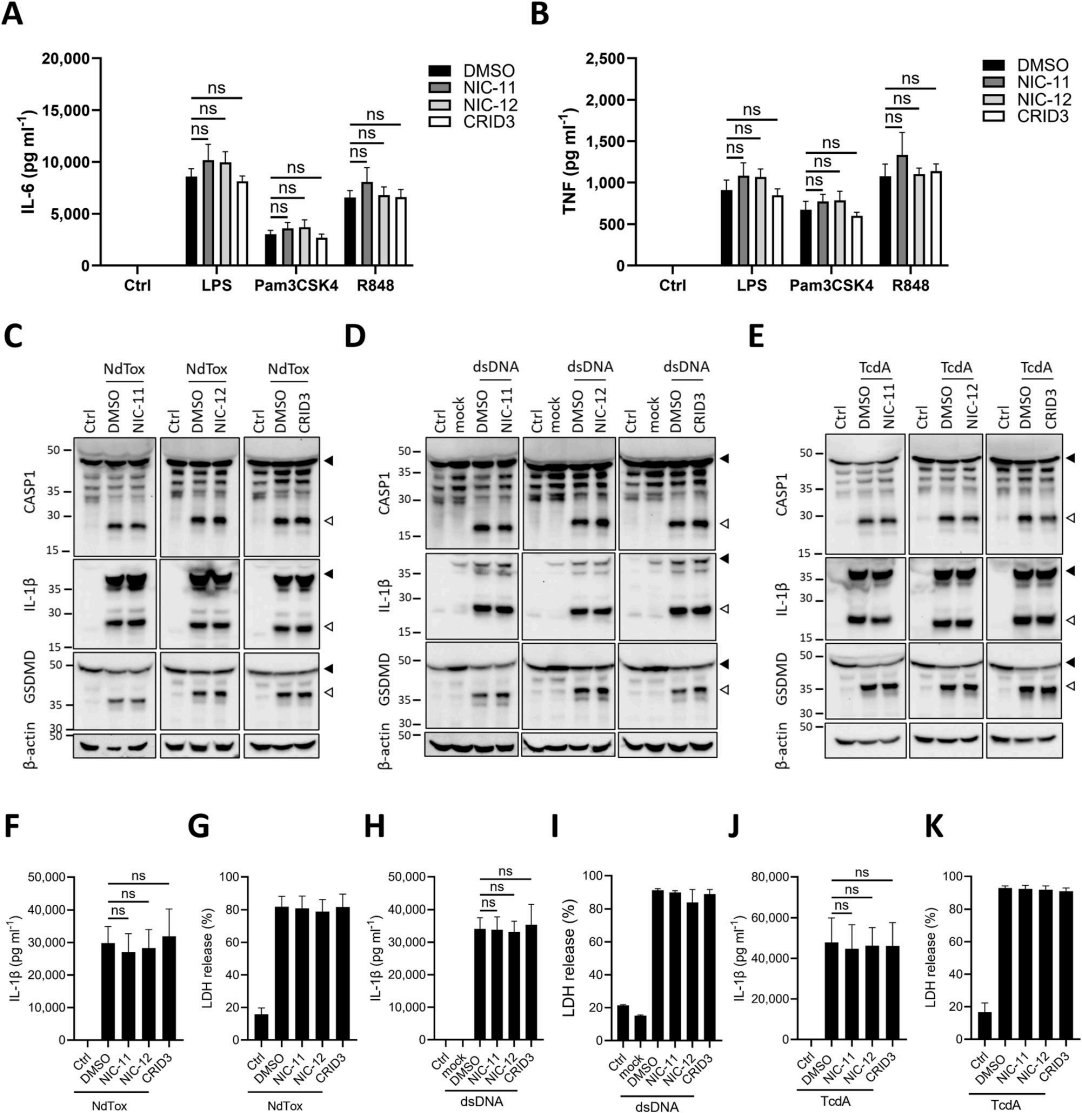
NIC-11 and NIC-12 target NLRP3 signaling upstream of caspase-1 in mouse BMDMs. **(A, B)** Primary wild-type BMDMs were treated with 0.01% DMSO or with 1 μM of the indicated compounds, before being either left untreated (Ctrl) or stimulated with LPS (100 ng ml^−1^), Pam3CSK4 (500 ng ml^−1^), or Resiquimod (2 μg ml^−1^). **(A, B)** Supernatants were analyzed for IL-6 (A) or TNF (B) secretion. Values are depicted as mean ± SEM of n = 3 biological replicates. Statistical significance was analyzed by two-way ANOVA with Bonferroni’s multiple comparisons test. ns, nonsignificant. **(C, D, E, F, G, H, I, J, K)** iBMDM were either left untreated (Ctrl) or primed with LPS (100 ng ml^−1^), followed by treatment with either 0.01% DMSO or 1 μM of the indicated compounds before being stimulated with NeedleTox (NdTox, 500 ng ml^−1^ PA and 100 ng ml^−1^ LFn-Needle) (C, F, G) or transfected with herring sperm dsDNA (2 μg ml^−1^) (D, H, I) or exposed to TcdA (1 μg ml^−1^) (E, J, K). **(C, D, E)** Lysates were immunoblotted for caspase-1, IL-1β, GSDMD, and β-actin. One representative Western blot is shown out of three independent experiments. Arrows indicate the unprocessed (black) and processed (white) forms, respectively. **(F, G, H, I, J, K)** Supernatants were analyzed for IL-1β secretion (F, H, J) or LDH activity (G, I, K). IL-1β secretion and LDH activity are depicted as mean ± SEM of n = 3 biological replicates. Statistical significance was analyzed using one-way ANOVA with Bonferroni’s multiple comparisons test. ns, nonsignificant.

**Figure S2.**
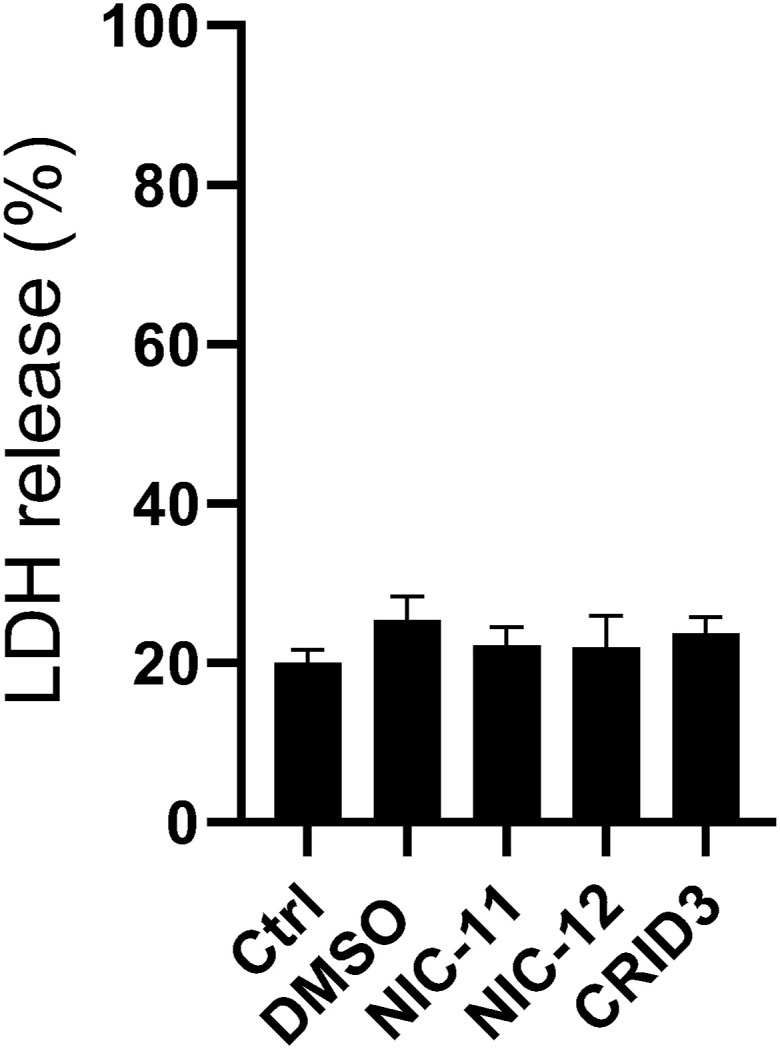
NIC-11 and NIC-12 do not induce cytotoxicity in BMDMs. Primary wild-type BMDMs were either left untreated (Ctrl) or treated with 0.1% DMSO or 10 μM of the indicated compounds for 24 h. Supernatants were analyzed for LDH activity. LDH activity is depicted as mean ± SEM of n = 3 biological replicates.

Activation of the NLRP3 inflammasome proceeds through a biphasic process in which NLRP3 activation is preceded by a priming step that serves to transcriptionally up-regulate NLRP3 and pro-IL-1ß expression via TLR-induced NF-κB signaling (Bauernfeind et al, 2009). Although in our previous experiments the inhibitors were provided after LPS priming, we confirmed that they do not target TLR-induced secretion of the NF-κB–dependent cytokines IL-6 and TNF (Fig 2A and B). Neither did the inhibitors induce cytotoxicity in BMDMs, even after 24 h exposure at a much higher dose (10 μM) than required for complete inhibition of the NLRP3 inflammasome (Fig S2).

Contrasting markedly to their potent inhibitory effects on NLRP3 stimuli (Fig 1), NIC-11 and NIC-12 failed to suppress cleavage of caspase-1, proIL-1ß, and GSDMD in LPS-primed immortalized BMDMs (iBMDMs) that were subsequently treated with NeedleTox (NdTox) for activation of the NLRC4 inflammasome or transfected with dsDNA to activate the AIM2 inflammasome (Fig 2C and D). Similar to CRID3, they also did not modulate these responses upon activation of the Pyrin inflammasome in TcdA-intoxicated iBMDMs (Fig 2E). Consistent herewith, secretion of IL-1ß and induction of pyroptosis by the NLRC4, AIM2, or Pyrin inflammasome pathways were unchanged in the presence of NIC-11, NIC-12, or CRID3 (Fig 2F–K). Collectively, these results show that NIC-11 and NIC-12 do not target NLRP3 priming and suggest that they selectively target the NLRP3 inflammasome pathway upstream of caspase-1.

### Selective NLRP3 inflammasome inhibition in primary human monocytes

**Figure 3.**
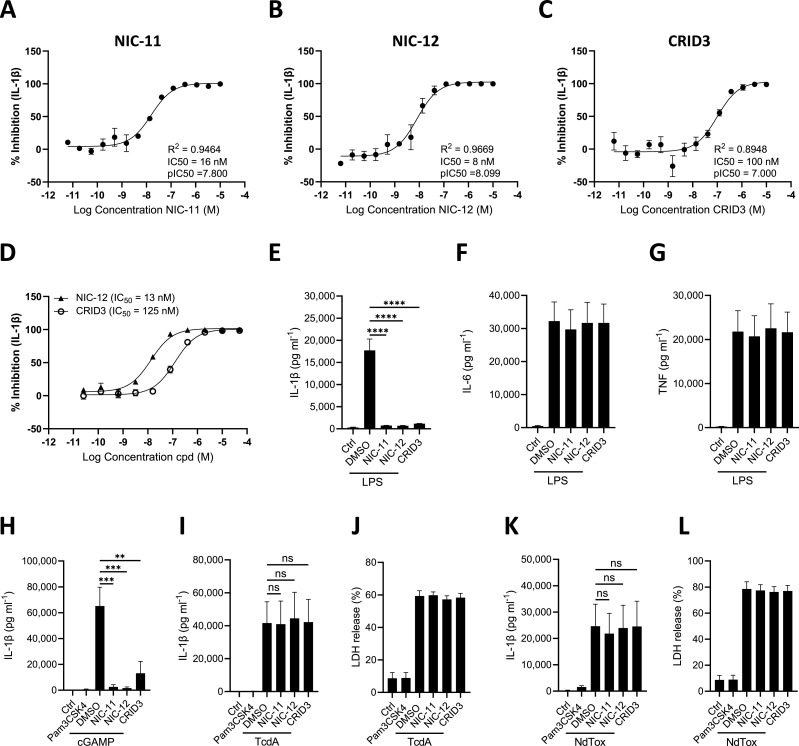
Selective NLRP3 inflammasome inhibition in primary human monocytes. **(A, B, C)** Dose–response curves of the effect of NIC-11 (A), NIC-12 (B), and CRID3 (C) on IL-1β secretion from LPS-stimulated (100 ng ml^−1^) primary human monocytes. IL-1β secretion is depicted as mean ± SEM of n = 3 biological replicates. **(D)** Dose–response curves of the effect of NIC-12 and CRID3 on IL-1β secretion from LPS-stimulated (100 ng ml^−1^) primary human PBMCs. IL-1β secretion is depicted as mean ± SEM of n = 5 biological replicates. **(E, F, G)** Primary human monocytes were exposed to 0.01% DMSO or 1 μM of the indicated compounds before being subjected to either no treatment (Ctrl) or treatment with LPS (100 ng ml^−1^). **(E, F, G)** Supernatants were analyzed for IL-1β (E), IL-6 (F), and TNF (G) secretion. **(H, I, J, K, L)** Primary human monocytes were either left untreated (Ctrl) or treated with Pam3CSK4 (100 ng ml^−1^), before treatment with 0.01% DMSO or 1 μM of the indicated compounds, followed by stimulation with 2'3'-cGAMP (50 μg ml^−1^) (H), TcdA (1 μg ml^−1^) (I, J), or NdTox (500 ng ml^−1^ PA and 100 ng ml^−1^ LFn-Needle) (K, L). **(H, I, J, K, L)** Supernatants were analyzed for IL-1β secretion (H, I, K) or LDH activity (J, L). Values are depicted as mean ± SEM of n = 3 biological replicates. Statistical significance was analyzed by one-way ANOVA with Bonferroni’s multiple comparisons test. ****P ≤ 0.0001; ***P ≤ 0.001; **P ≤ 0.01; ns, nonsignificant.

To extend our findings to the human system, we next examined the potency and selectivity profiles of NIC-11 and NIC-12 in human primary blood monocytes. In contrast to mouse macrophages, TLR4 stimulation by LPS alone induces NLRP3-dependent secretion of IL-1ß in primary human blood monocytes (Netea et al, 2009; O’Brien et al, 2017). NIC-11 and NIC-12 showed potent activity in LPS-stimulated blood monocytes and dose-dependently inhibited IL-1ß secretion with IC_50_ values of 16 and 8 nM, respectively (Fig 3A and B). The IC_50_ value of CRID3 in this assay was 100 nM (Fig 3C), indicating that NIC-11 and NIC-12 display potencies in human blood monocytes that are, respectively, 6 and 12.5 times better than that of the reference NLRP3 inhibitor CRID3. Similar results were obtained with LPS-stimulated human PBMCs, where NIC-12 exhibits a tenfold higher efficacy compared with CRID3 (Fig 3D). Moreover, each of the three inhibitors abolished LPS-induced IL-1ß secretion from human blood monocytes when used at 1 μM without modulating LPS-induced IL-6 and TNF secretion levels (Fig 3E–G), demonstrating that NIC-11 and NIC-12, like CRID3, suppress LPS-induced NLRP3 activation downstream of TLR4-induced NF-κB signaling. Their inhibitory activity against NLRP3 signaling is not limited to LPS as a stimulus because activation of the NLRP3 inflammasome by the endogenous STING agonist cGAMP (Gaidt et al, 2017) was also blocked by NIC-11 and NIC-12 pretreatment (Fig 3H). Contrastingly, NIC-11, NIC-12, and CRID3 did not inhibit IL-1ß secretion and pyroptosis induction in human monocytes treated with TcdA for activation of the Pyrin inflammasome or NdTox for activation of the NLRC4 inflammasome (Fig 3I–L). Together, these results demonstrate that NIC-11 and NIC-12 potently and selectively inhibit activation of the NLRP3 inflammasome in human monocytes.

### Target engagement assays reveal NLRP3 as the physical target of NIC-11 and NIC-12

**Figure S3.**
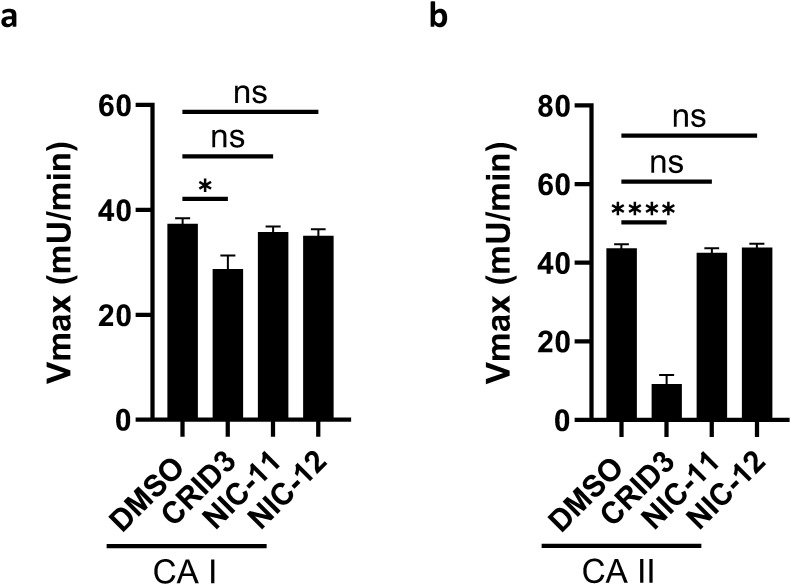
Carbonic anhydrases I (CA I) and CA II are off-targets of CRID3 but not of NIC-11 and NIC-12. **(A, B)** CA I (A) or CA II (B) were preincubated with 1% DMSO or 100 μM of the indicated compounds before incubation with the para-nitrophenol acetate (p-NPA) substrate. Esterase activity was measured by quantifying the formation of 4-nitrophenol at 405 nm. Catalytic activity, expressed as Vmax, represents the mean ± SEM of three biological repeats. Statistical significance was analyzed using one-way ANOVA with Bonferroni’s multiple comparisons test.

Our functional studies above suggest that NIC-11 and NIC-12 inhibit NLRP3 inflammasome activation apically in the pathway at the level of NLRP3 itself or an essential cofactor. Recent studies provided robust evidence from biochemical (Coll et al, 2019; Tapia-Abellan et al, 2019; Vande Walle et al, 2019) and structural biology (Dekker et al, 2021; Hochheiser et al, 2022; McBride et al, 2022) studies that CRID3 targets a pocket in the central NACHT domain of inactive NLRP3. In addition, CRID3 was recently shown to noncompetitively inhibit the esterase activity of carbonic anhydrase II with micromolar potency (IC_50_ = 11 μM) (Kennedy et al, 2021). We confirmed this off-target activity of CRID3 in our studies and observed significant inhibition of the related enzyme carbonic anhydrase I as well (Fig S3A and B). In marked contrast, neither NIC-11 nor NIC-12 displayed these off-target effects on carbonic anhydrases I and II (Fig S3A and B).

**Figure 4.**
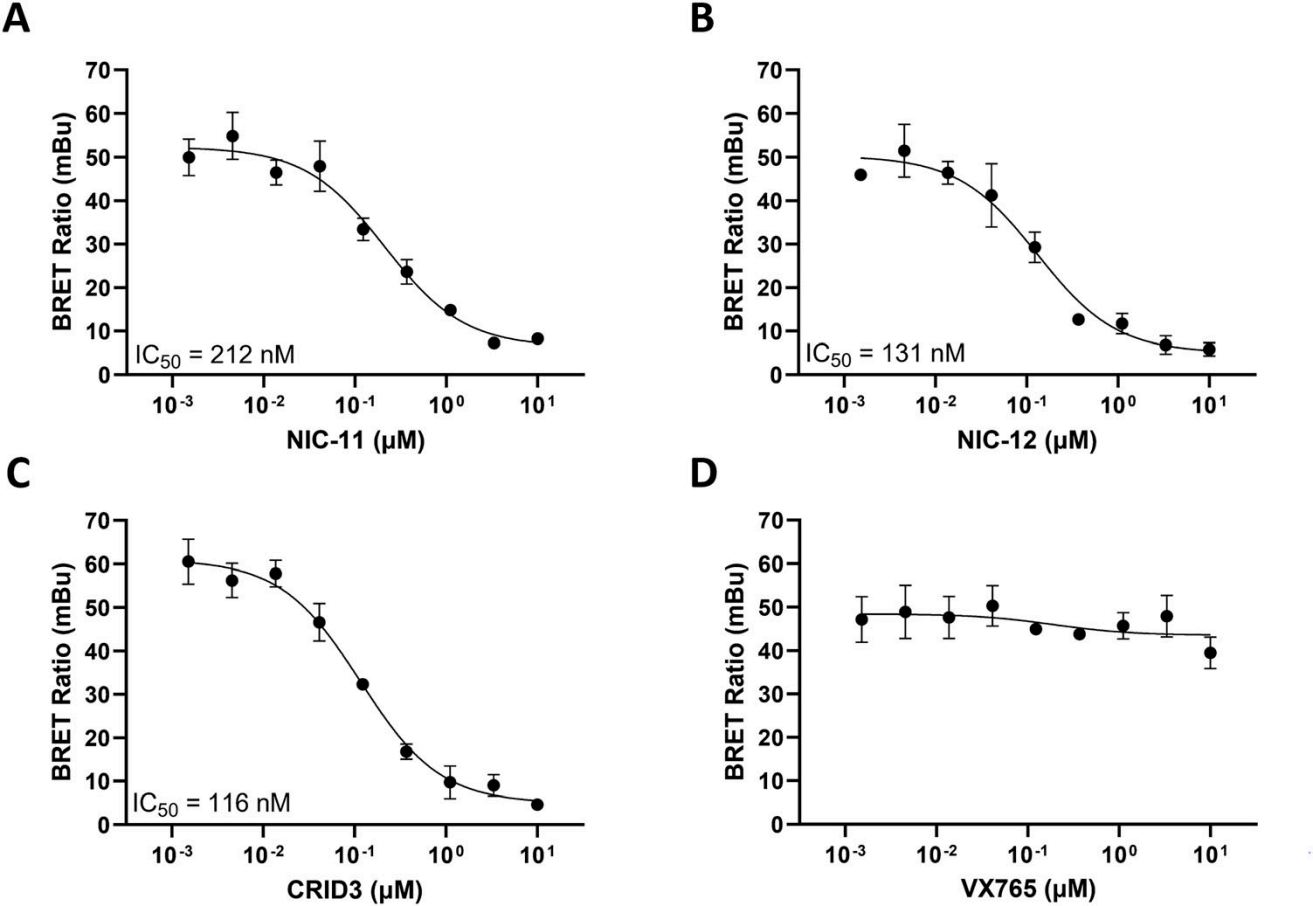
NanoBRET target engagement assays reveal direct binding of NIC-11 and NIC-12 to the NLRP3 NACHT domain. **(A, B, C, D)** Intracellular NLRP3-Nanoluc fusion protein was incubated with serially diluted NIC-11 (A), NIC-12 (B), CRID3 (C), or the caspase-1 inhibitor VX765 (D) in the absence or presence of 0.16 μM of the NanoBRET NLRP3 tracer. The BRET ratio is depicted as mean ± SEM of n = 3 biological repeats.

To explore the hypothesis that NIC-11 and NIC-12 may inhibit the NLRP3 inflammasome by targeting NLRP3 directly, we used a novel NLRP3 NanoBRET assay that allows to quantify the apparent affinity of test compounds for binding to ectopically expressed NLRP3 in live cells (Teske et al, 2024). The assay works by generating a bioluminescence resonance energy transfer (BRET) signal when a NLRP3/Nanoluc fusion construct expressed in HEK293 cells interacts with a cell-permeable, fluorescent small-molecule NLRP3 binder (called “NLRP3 tracer”). Test compounds that bind to the NLRP3 NACHT may disrupt the interaction between the NLRP3/Nanoluc fusion protein and the NLRP3 tracer, resulting in a dose-dependent inhibition of the NanoBRET signal (Teske et al, 2024). Notably, our analyses indicated that NIC-11 and NIC-12 both suppressed the NanoBRET signal in a dose-dependent manner with IC_50_ values of 212 and 131 nM, respectively (Fig 4A and B). As expected, CRID3 dose-dependently inhibited the BRET signal, whereas the cell-permeable caspase-1 inhibitor VX765 had no effect (Fig 4C and D), demonstrating specificity of these findings.

### Structural modeling reveals unique binding mode in the CRID3-binding pocket of NLRP3

**Figure S4.**
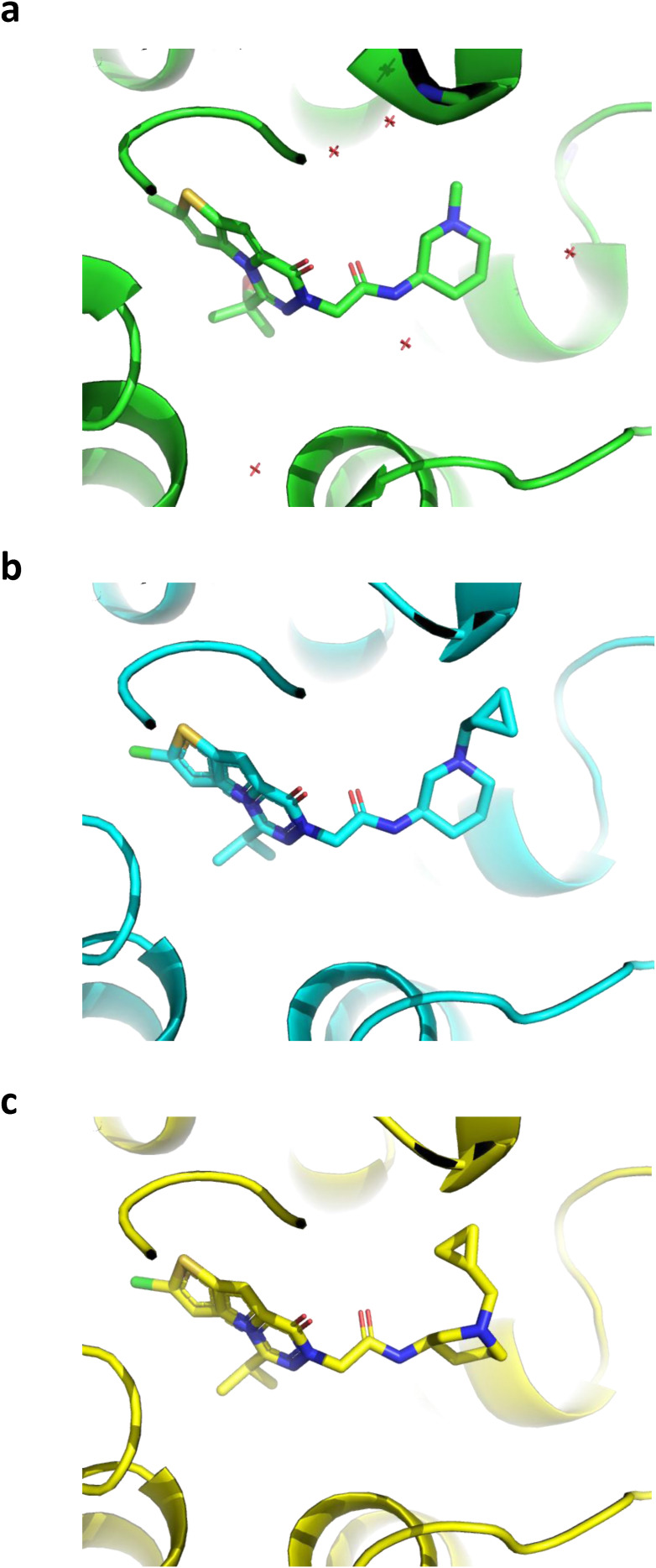
Exemplar structural model images. **(A, B, C)** Exemplar images of the NLRP3 NACHT structure bound to NP3-562 (PDB: 8RI2) (A), which was used as a template to build a model of the NIC-12–bound NLRP3 NACHT structure (B, C). To rebuild the ligand, the isopropanol moiety was replaced by an isopropyl group and the N-methyl group was replaced by N-methylcyclopropyl. After minimization, strain energy analysis indicated that the N-methylcyclopropyl group of NIC-12 prefers to adopt an axial orientation. **(C)** Thus, the group was rebuilt using an axial orientation and minimized for the final model (C).

**Figure 5.**
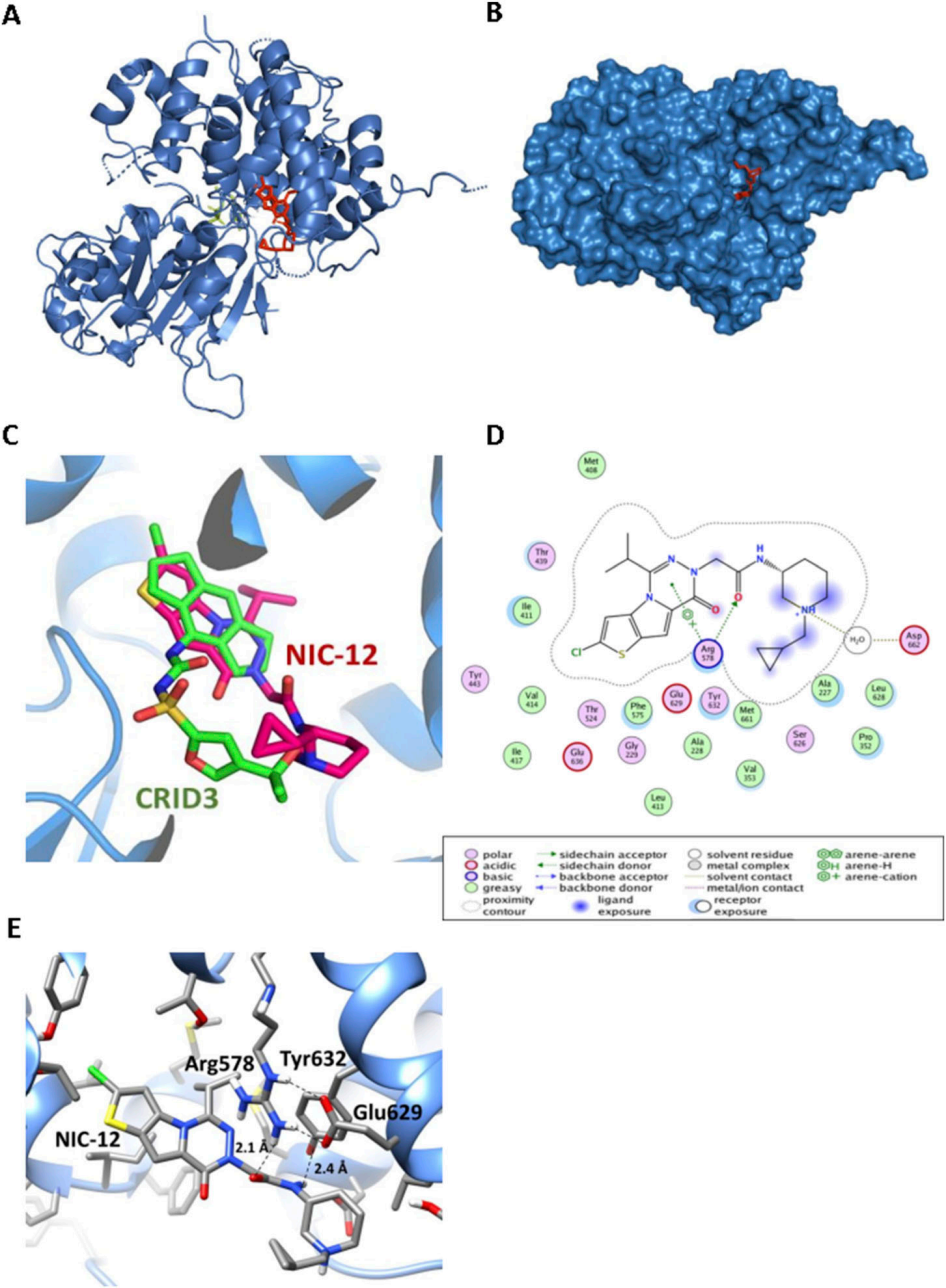
Structural modeling reveals unique binding mode in the CRID3-binding site. **(A)** Model of NIC-12 (red) bound to NLRP3 NACHT (blue) and ADP (yellow). **(B)** Surface map of NLRP3 NACHT displaying the axial conformation of the piperidine cyclopropylmethyl side chain of NIC-12 in the binding pocket. **(C)** Comparison between binding pose of CRID3 (in green) and NIC-12 (in red) in the NLRP3 binding pocket. **(D)** Overview of NIC-12 binding interactions with the NLRP3 NACHT. Color codes are as defined in the box below the interaction map. **(E)** Close-up of the NIC-12 binding site depicting interactions between the side chains of R578 and Y632 with the central amide of NIC-12.

During the revision process of our manuscript, a new X-ray structure was reported detailing the NLRP3 NACHT domain in complex with NP3-562, a compound that is chemically related to NIC-11 and NIC-12 (Velcicky et al, 2024). To model the binding interactions between NIC-12 and the NLRP3 NACHT domain, this structure was used as a template to build a model of the NIC-12–bound structure. To rebuild the ligand, the isopropanol moiety of NP3-562 (Fig S4A) was replaced by an isopropyl group and the N-methyl group was replaced by the N-methylcyclopropyl group of NIC-12. The Merck molecular force field (MMFF94x) was then used to minimize the ligand while keeping the protein, water, and ADP ligand fixed (Fig S4B). Examination of the strain energy indicated that the N-methylcyclopropyl group prefers to adopt an axial orientation. Thus, the group was rebuilt using an axial orientation and minimized again (Figs 5A and S4C).

NIC-12 binds to the recently discovered CRID3-binding pocket in the NLRP3 NACHT (Dekker et al, 2021; Hochheiser et al, 2022; McBride et al, 2022) but in a critically different conformation compared with CRID3 (Fig 5B and C). A comparison between our model of NLRP3 bound to NIC-12 and the recently reported NLRP3/CRID3 structures (Dekker et al, 2021; Hochheiser et al, 2022; McBride et al, 2022) indicates that the hexahydro-s-indacene group of CRID3 binds in the same hydrophobic cavity of NIC-12 (Fig 5C). In the case of NIC-12, this hydrophobic cavity is occupied by the thieno[2′,3′:4,5]pyrrole[1,2-d][1,2,4]triazine core moiety of NIC-12 and surrounded by F410, I411, F575, T439, and Y443 sidechains of the NLRP3 NACHT (Fig 5D). Interestingly, from there, the two inhibitors depart at 45° from each other to engage two distinct surfaces of NLRP3 but converging again toward the opening of the allosteric pocket that is connected to the bulk solvent (Fig 5C). NIC-12’s central amide group interacts with R578 and Y632 (Fig 5E), whereas the central sulfonylurea linker of CRID3 is anchored by the Walker A motif, R351, and R578 (Dekker et al, 2021; Hochheiser et al, 2022; McBride et al, 2022). Finally, the distal cyclobutane-substituted piperidine moiety of NIC-12 extends toward the solvent area, reminiscent of the externally oriented isopropyl furan moiety of CRID3 toward the LRR region (Fig 5D and E). Collectively, our model suggests that NIC-11 and NIC-12 inhibit NLRP3 signaling by physically targeting the recently identified CRID3-binding pocket in a critically different conformation.

### NIC-11 and NIC-12 exhibit increased potency in MWS-associated Nlrp3^A350V^ BMDMs

Gain-of-function mutations in the *NLRP3* gene, which lead to the protein being continuously active, result in a spectrum of dominantly inherited conditions known as CAPS in patients (Hoffman et al, 2001). Unlike wild-type macrophages, BMDMs expressing the MWS-associated Nlrp3^A350V^ mutant secrete significant levels of IL-1β into their culture medium upon LPS stimulation alone (Brydges et al, 2009). We previously reported that 1 μM CRID3 abolished LPS-induced IL-1ß secretion from wild-type and Nlrp3^A350V^ mutant BMDMs, but this concentration was ineffective in inhibiting IL-1ß secretion from LPS+nigericin-stimulated Nlrp3^A350V^ BMDMs (Vande Walle et al, 2019).

**Figure S5.**
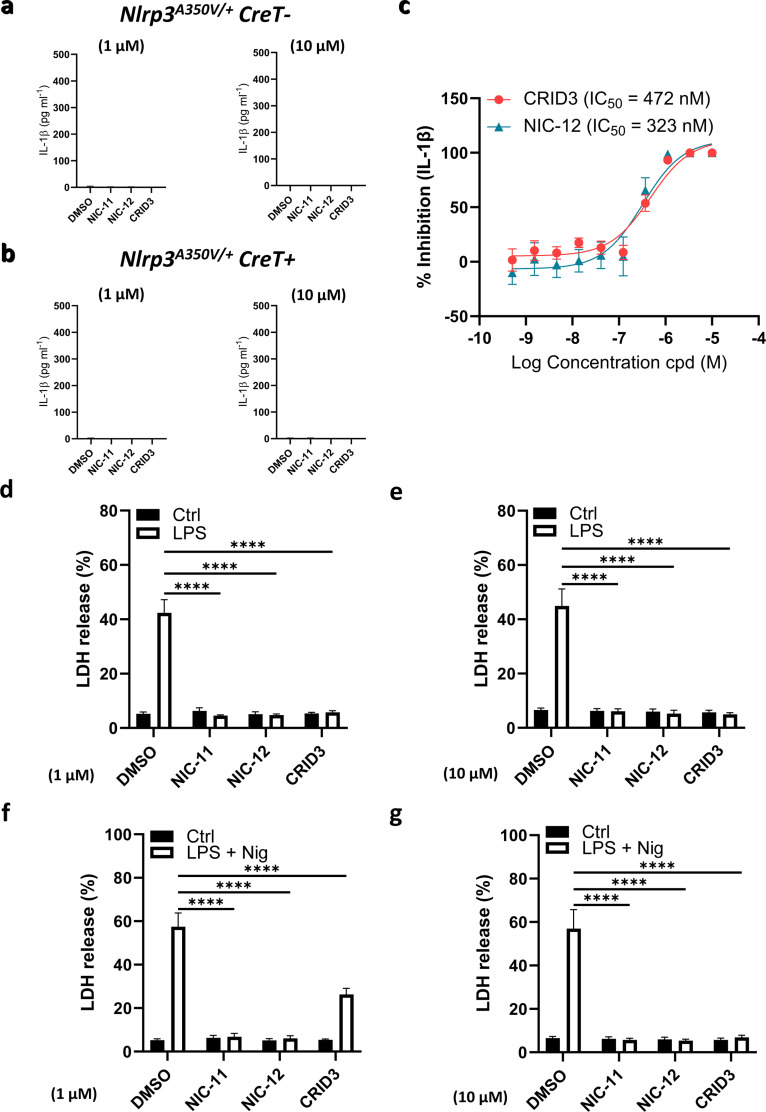
NIC-11 and NIC-12 inhibit pyroptosis of MWS-associated Nlrp3^A350V^ BMDMs with increased potency. **(A, B)** BMDMs from tamoxifen-treated *Nlrp3*^*A350V/+*^*CreT*− (A) or *Nlrp3*^*A350V/+*^*CreT+* (B) mice were stimulated with DMSO (0.1% or 0.01%) or the indicated compounds at 1 or 10 μM. Supernatants were analyzed for IL-1β secretion. All data represent mean ± SEM of n = 3 biological repeats. **(C)** Dose–response curves of the effect of NIC-12 and CRID3 on IL-1β secretion from LPS-stimulated (100 ng ml^−1^) BMDMs from tamoxifen-treated *Nlrp3*^*A350V/+*^*CreT+* mice. IL-1β secretion is depicted as mean ± SEM of n = 4 biological replicates. **(D, E, F, G)** BMDMs from tamoxifen-treated *Nlrp3*^*A350V/+*^*CreT+* mice were either stimulated with 0.01% DMSO or 1 μM (D, F) or 0.1% DMSO or 10 μM (E, G) of the indicated compounds before treatment with LPS (100 ng ml^−1^) (D, E) or LPS + nigericin (10 μM) (F, G). Supernatants were analyzed for secreted IL-1ß or LDH activity, as indicated. IL-1β secretion and LDH release are depicted as mean ± SEM of n = 3 biological repeats. Statistical significance was analyzed using two-way ANOVA with Bonferroni’s multiple comparisons test. ****P ≤ 0.0001.

**Figure 6.**
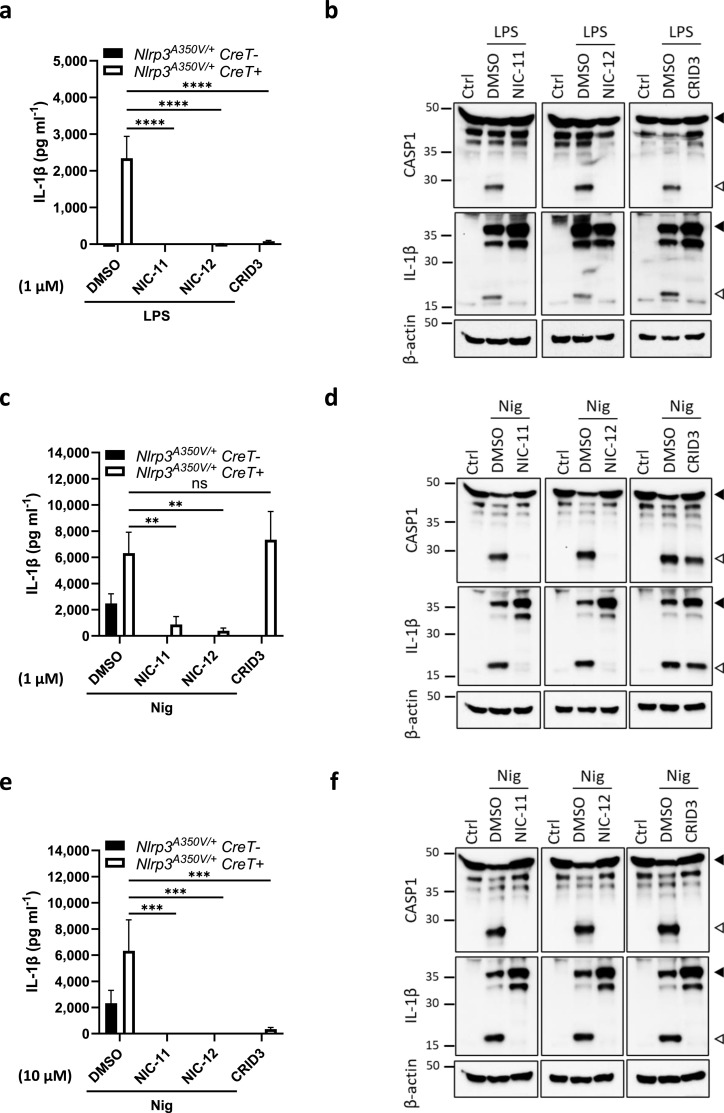
NIC-11 and NIC-12 exhibit increased potency in MWS-associated Nlrp3^A350V^ BMDMs. **(A)** BMDMs from tamoxifen-treated *Nlrp3*^*A350V/+*^*CreT*− or *Nlrp3*^*A350V/+*^*CreT+* mice were either stimulated with 0.01% DMSO or 1 μM of the indicated compounds before treatment with LPS (100 ng ml^−1^). Supernatants were analyzed for IL-1β secretion. **(B)** BMDMs from tamoxifen-treated *Nlrp3*^*A350V/+*^*CreT+* mice were either left untreated (Ctrl) or treated with either 0.01% DMSO or 1 μM of the indicated compounds before treatment with LPS (100 ng ml^−1^). Lysates were immunoblotted for caspase-1, IL-1β, and β-actin. **(C)** BMDMs from tamoxifen-treated *Nlrp3*^*A350V/+*^*CreT*− or *Nlrp3*^*A350V/+*^*CreT+* mice were either stimulated with 0.01% DMSO or 1 μM of the indicated compounds before treatment with LPS + nigericin (Nig, 10 μM). Supernatants were analyzed for IL-1β secretion. **(D)** BMDMs from tamoxifen-treated *Nlrp3*^*A350V/+*^*CreT+* mice were either left untreated (Ctrl) or treated with either 0.01% DMSO or 1 μM of the indicated compounds before treatment with LPS + nigericin (10 μM). Lysates were immunoblotted for caspase-1, IL-1β, and β-actin. **(E)** BMDMs from tamoxifen-treated *Nlrp3*^*A350V/+*^*CreT*− or *Nlrp3*^*A350V/+*^*CreT+* mice were either stimulated with 0.1% DMSO or 10 μM of the indicated compounds before treatment with LPS + nigericin (Nig, 10 μM). Supernatants were analyzed for IL-1β secretion. **(F)** BMDMs from tamoxifen-treated *Nlrp3*^*A350V/+*^*CreT+* mice were either left untreated (Ctrl) or treated with either 0.1% DMSO or 10 μM of the indicated compounds before treatment with LPS + nigericin (10 μM). Lysates were immunoblotted for caspase-1, IL-1β, and β-actin. IL-1β secretion is depicted as mean ± SEM of n = 3 biological replicates. Statistical significance was analyzed using two-way ANOVA with Bonferroni’s multiple comparisons test. ****P ≤ 0.0001; ***P ≤ 0.001; **P ≤ 0.01; ns, nonsignificant. One representative Western blot is shown out of three independent experiments. Arrows indicate the unprocessed (black) and processed (white) forms, respectively.

To determine whether this also holds true for NIC-11 and NIC-12, we bred mice that are homozygous for the *Nlrp3*^A350V^ allele to transgenic mice that hemizygously express the tamoxifen-inducible Cre-ERT2 fusion gene (*CreT*) (Ventura et al, 2007). After tamoxifen treatment and excision of the floxed neomycin resistance cassette, BMDMs of the resulting *Nlrp3*^*A350V/+*^*CreT*^*+*^ mice express NLRP3 from both the wild-type and the mutant *Nlrp3*^A350V^ alleles. BMDMs from CreT-negative littermates (*Nlrp3*^*A350V/+*^*CreT*^*−*^), which only express NLRP3 from the wild-type allele, were used as controls in these experiments. As a control, we confirmed that neither NIC-11, NIC-12, nor CRID3 alone triggered IL-1β secretion from *Nlrp3*^*A350V/+*^*CreT*^*−*^ or *Nlrp3*^*A350V/+*^*CreT*^*+*^ macrophages when dosed at a final concentration of 1 or 10 μM (Fig S5A and B). As reported (Vande Walle et al, 2019), *Nlrp3*^*A350V/+*^*CreT*^*+*^ macrophages exhibited high levels of secreted IL-1β in response to LPS stimulation alone (Fig 6A). This was accompanied by prominent maturation of caspase-1 and proIL-1β as demonstrated by immunoblot analysis (Fig 6B). As expected, no IL-1β secretion was observed from LPS-stimulated *Nlrp3*^*A350V/+*^*CreT*^*−*^ BMDMs that express wild-type Nlrp3 only (Fig 6A). A dose–response analysis comparing NIC-12 and CRID3 suggested that both potently inhibited LPS-induced IL-1β secretion from *Nlrp3*^*A350V/+*^*CreT*^*+*^ macrophages with associated IC_50_ values of 323 and 472 nM, respectively (Fig S5C). In agreement, 1 μM of NIC-11, NIC-12, or CRID3 abolished LPS-induced IL-1β secretion (Fig 6A) and pyroptosis induction in LPS-stimulated *Nlrp3*^*A350V/+*^*CreT*^*+*^ macrophages (Fig S5D and E). Correspondingly, this concentration of NIC-11, NIC-12, and CRID3 inhibited Nlrp3^A350V^-driven cleavage of caspase-1 and IL-1β in lysates of LPS-stimulated *Nlrp3*^*A350V/+*^*CreT*^*+*^ macrophages (Fig 6B).

Unlike LPS, nigericin potently triggered IL-1β secretion, pyroptosis and maturation of caspase-1 and proIL-1β from both *Nlrp3*^*A350V/+*^*CreT*^*+*^ and *Nlrp3*^*A350V/+*^*CreT*^*−*^ control BMDMs (Fig 6C). Although 1 μM CRID3 abolished nigericin-induced IL-1β secretion from *Nlrp3*^*A350V/+*^*CreT*^*−*^ control BMDMs, it failed to inhibit IL-1β secretion from nigericin-treated *Nlrp3*^*A350V/+*^*CreT*^*+*^ BMDMs (Fig 6C). Furthermore, nigericin-induced pyroptosis in *Nlrp3*^*A350V/+*^*CreT*^*+*^ macrophages was only partially suppressed by 1 μM CRID3 (Fig S5F). Consistent with these results, cleavage of caspase-1 and proIL-1β in nigericin-stimulated *Nlrp3*^*A350V/+*^*CreT*^*+*^ BMDMs was not inhibited by 1 μM CRID3 (Fig 6D), and pretreatment with 10 μM CRID3 was required to effectively abrogate the aforementioned inflammasome responses (Figs 6E and F and S5G). In marked contrast, 1 μM of either NIC-11 or NIC-12 was sufficient to blunt IL-1β secretion (Fig 6C), pyroptosis (Fig S5F), and cleavage of caspase-1 and proIL-1β (Fig 6D) in nigericin-stimulated *Nlrp3*^*A350V/+*^*CreT*^*+*^ BMDMs. Together, these results demonstrate that NIC-11 and NIC-12 exhibit increased potency in an ex vivo mouse model of CAPS.

### Activity in CAPS patient PBMCs and in the in vivo mouse model of endotoxemia

**Figure 7.**
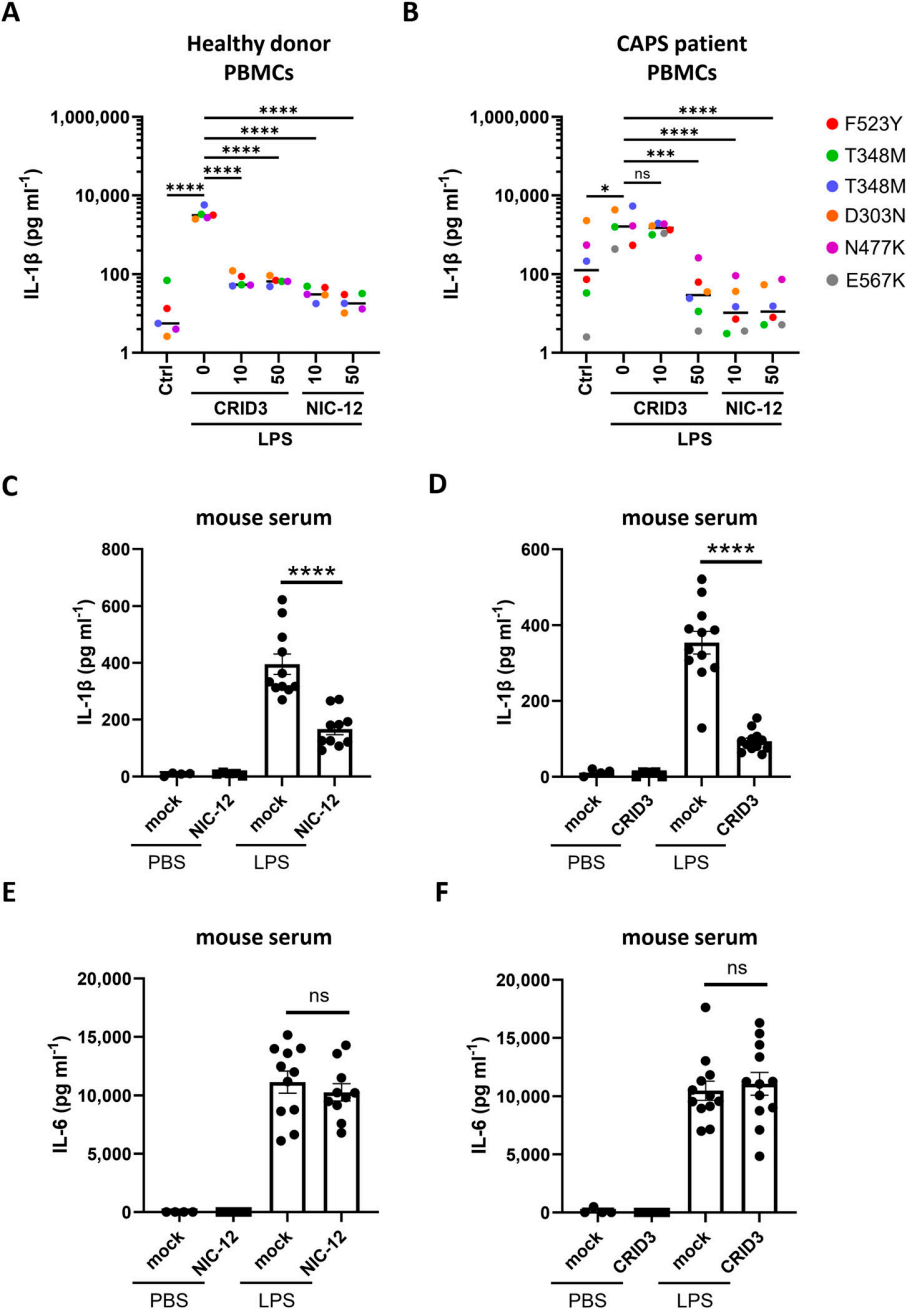
Activity in CAPS patient PBMCs and in an in vivo mouse model of endotoxemia. **(A, B)** PBMCs from healthy donors (n = 5) (A) and CAPS patients (n = 6) (B) were exposed to 0.1% DMSO or the indicated concentrations of CRID3 or NIC-12 before being subjected to either no treatment (Ctrl) or treatment with LPS (100 ng ml^−1^). Supernatants were analyzed for IL-1β secretion. **(C, D, E, F)** Wild-type C57BL/6J mice were pretreated with vehicle (mock) or the indicated compounds (50 mg kg^−1^) and subsequently challenged with either PBS or LPS (10 mg kg^−1^). Each dot represents an individual mouse. PBS recipient group: mock, n = 4; NIC-12 or CRID3, n = 5; LPS recipient group: mock, n = 11–12; NIC-12 or CRID3, n = 10–12. **(C, D, E, F)** Serum levels of IL-1β (C, D) and IL-6 (E, F) were analyzed. Cytokine analysis is depicted as mean ± SEM. Statistical significance was analyzed using one-way ANOVA with Bonferroni’s multiple comparisons test. ****P ≤ 0.0001; ***P ≤ 0.001; *P ≤ 0.05; ns, nonsignificant.

Our results in *Nlrp3*^A350V^-expressing mouse macrophages suggest that NIC-11 and NIC-12 may exhibit increased potency against CAPS mutations. To examine how this translates to the human context, we collected PBMCs of five healthy donors as controls and six patients with genetically defined CAPS mutations (F523Y, T348M, D303N, N477K, and E567K) that have been assigned a “pathogenic” or “likely pathogenic” status in InFevers—a manually curated reference database for variants in autoinflammatory disease-associated genes (Van Gijn et al, 2018). LPS stimulation triggered significant IL-1ß secretion from healthy donor PBMCs, and 10 μM NIC-12 or CRID3 completely abolished LPS-induced IL-1ß secretion from healthy donor PBMCs (Fig 7A). Unstimulated CAPS PBMCs already secreted substantial levels of IL-1ß, and this was further elevated by LPS stimulation (Fig 7B). Notably, pretreatment with 10 μM CRID3 did not alter IL-1ß secretion from LPS-stimulated PBMCs of the six CAPS donors in our cohort, and a dose of 50 μM CRID3 was required for effective suppression (Fig 7B). In marked contrast, pretreatment with 10 μM NIC-12 sufficed to abolish IL-1ß secretion in CAPS PBMCs (Fig 7B). Collectively, these results demonstrate that NIC-12 exhibits increased potency against various CAPS mutations.

We finally set out to compare the effects of NIC-12 and CRID3 in LPS-induced endotoxemia, an in vivo disease model that mimics various aspects of acute septic shock in patients, including elevated cytokine production and extensive leukocyte apoptosis (Lamkanfi et al, 2010). Previous work established that NLRP3 is a critical driver of increased IL-1ß levels in serum of LPS-challenged mice, whereas caspase-11 activation promotes lethality in LPS-induced endotoxemia (Kayagaki et al, 2011). As expected, intraperitoneal administration of LPS significantly increased circulating levels of IL-1ß (Fig 7C and D) and IL-6 (Fig 7E and F) as measured 3 h post-LPS challenge. Neither NIC-12 nor CRID3 changed basal levels of circulating IL-1ß (Fig 7C and D) and IL-6 (Fig 7E and F) in unchallenged animals. However, the two NLRP3 inhibitors significantly diminished circulating levels of IL-1ß in LPS-challenged mice (Fig 7C and D) without interfering with circulating IL-6 measurements (Fig 7E and F). Although we did not observe statistically significant differences in potency between CRID3 and NIC-12, there might be a trend toward CRID3 exhibiting slightly better inhibition of NLRP3 in this in vivo disease setting. Regardless, these findings suggest that both CRID3 and NIC-12 selectively inhibit NLRP3 activation in a preclinical in vivo disease model of acute shock.

## Discussion

The NLRP3 inflammasome contributes critically to detrimental inflammation and immune-mediated tissue damage in autoinflammatory, autoimmune, and immunometabolic and neurodegenerative diseases (Van Opdenbosch & Lamkanfi, 2019; Voet et al, 2019). CAPS and other autoinflammatory disease patients are currently treated with biologics that target secreted IL-1. However, chronic IL-1-blockade increases risk for fatal infections and sepsis, suggesting that upstream targeting of the NLRP3 inflammasome may potentially be a safer and more efficacious therapeutic strategy as it would block production of the central inflammatory mechanisms that contribute to the pathology while at the same time keeping non-targeted inflammasomes available to produce IL-1β to cope with infections (Ridker et al, 2017). Hence, there is substantial interest in developing selective and potent NLRP3-targeted inhibitors for the treatment of CAPS and other diseases (Vande Walle & Lamkanfi, 2024).

Various compounds that inhibit the NLRP3 inflammasome have been reported in the literature, and several are currently in early human studies (Vande Walle & Lamkanfi, 2024). However, limited insight in their molecular targets and mechanism of action puts a constraint on the development of potent and selective next-generation NLRP3 inflammasome inhibitors. Many of the clinical-grade NLRP3 inhibitors currently under investigation are chemically related to CRID3 (Vande Walle & Lamkanfi, 2024). The molecular target and binding pocket of the sulfonylurea NLRP3 inhibitor CRID3 has recently been discovered (Dekker et al, 2021; Hochheiser et al, 2022; McBride et al, 2022), shedding much needed light on the mechanism of action of this and structurally related NLRP3 inhibitors. However, a concern that persists with CRID3 is that its early clinical development was reportedly halted in phase 2 clinical studies because of high CRID3 dosing in healthy volunteers causing drug-induced liver injury (Shah et al, 2015; Kennedy et al, 2021). Although the exact reason for liver toxicity remains uncertain, there is suspicion that the metabolism of the isopropyl furan moiety of CRID3 may be a potential contributor to liver toxicity. In clinical-grade NLRP3 inhibitors based on CRID3, the toxicogenic furan has been substituted with alternative ring systems. These include the substituted pyrazol in emlenoflast, the piperidine moiety in RG6418 (Selnoflast), and the substituted pyrrolidine in the case of ZYIL1 (Vande Walle & Lamkanfi, 2024). Nevertheless, the discovery of additional inhibitor series with differentiated chemistry, high selectivity and potency, and a well-defined mechanism of action is urgently needed to advance the development of next-generation NLRP3-targeted inhibitors for clinical use.

Here, we report on two representative compounds from a novel class of pyrolo-triazine acetamide compounds, which we designated as NLRP3-inhibiting compounds (NIC)-11 and NIC-12. Both inhibitors lack the characteristic sulfonylurea found in CRID3. Instead of CRID3’s toxicogenic furan moiety, one contains a pyrimidine sidechain, whereas the other features a substituted piperidine on the apical side of the molecule. This implies that liver toxicity resulting from furan metabolism may not pose a significant concern with these inhibitors. We showed that NIC-11 and NIC-12 selectively inhibited inflammasome activation by various NLRP3 stimuli with nM potency in both human blood monocytes and mouse BMDMs without targeting NF-κB–dependent priming or activation of the NLRC4, AIM2, and Pyrin inflammasomes. Making use of BRET and structural modeling approaches, we identified NLRP3 as the physical target and mapped the interaction site to the recently discovered (Dekker et al, 2021; Hochheiser et al, 2022; McBride et al, 2022) binding pocket of CRID3 in the central NACHT domain of NLRP3. Given the shared binding site, it is likely that NIC-12 inhibits NLRP3 via the same mechanism of action as CRID3. Intriguingly, a comparison with the binding conformations of CRID3 and NIC-12 revealed that the externally oriented regions of the inhibitors exhibit notably distinct binding conformations relative to each other. An interesting prospect arises from this observation: it may serve as inspiration for the development of novel NLRP3 inhibitors that more effectively occupy the available binding space, potentially leading to further enhancements in potency and/or selectivity.

Regarding the latter, we showed that unlike CRID3, NIC-11 and NIC-12 lack off-target activity on carbonic anhydrases I and II. Finally, we demonstrate that NIC-12 suppresses circulating IL-1ß levels in vivo in LPS-challenged mice and inhibits NLRP3 inflammasome activation in CAPS monocytes and mouse macrophages expressing various disease-associated NLRP3 mutants with increased potency compared with CRID3. This study complements existing CRID3-based NLRP3 inhibitors by unveiling a new chemical class of highly potent and selective NLRP3-targeted inhibitors with a well-defined molecular mechanism that are devoid of carbonic anhydrase off-target effects, which should prove an invaluable tool in various NLRP3-associated human and murine pharmacological models. Furthermore, our results may have significant therapeutic ramifications for the treatment of CAPS, sepsis, atherosclerosis, Parkinson’s disease, and many other diseases in which excessive NLRP3 inflammasome activation contributes to pathology.

## Materials and Methods

### Chemical synthesis of NIC-11 and NIC-12

The synthesis, characterization, and purification of NIC-12 are described and depicted in Fig S1A and that of NIC-11 in Fig S1B. Both NIC-11 and NIC-12 (Supplemental Data 2) were purified to levels >95%. All reagents were purchased from commercial sources (Merck and TCI Europe) and used without further purification. The ^1^H and ^13^C NMR spectra were measured on various Bruker Avance spectrometers at room temperature, and data are reported as follows: the chemical shift (ppm units) from an internal standard, multiplicity (s, singlet; d, doublet; dd, double doublet; t, triplet; q, quartet; m, multiplet; and br, broad, spt septuplet), coupling constant *J* (Hz), and integration. High-resolution mass spectrometry analyses were performed using electrospray ionization in a positive ion modus on the Orbitrap instrument. HPLC was run on Vanquish UPLC (Thermo Fisher Scientific), MS Q-Exactive Plus (Thermo Fisher Scientific) instruments with a Kinetex C18 50 × 2.1-mm × 2.6-μm (Phenomenex) column used for HRMS. The IR spectra were recorded as films on a Perkin Elmer FT-IR Spectrum 1000 spectrometer. TLC was performed with Merck Silica gel, and 60–120 mesh silica gel was used for column chromatography.

Supplemental Data 2. NMR spectra of NIC-11 and NIC-12.

### Human PBMC and monocyte isolation

Blood samples were obtained from both healthy individuals (buffy coat) and CAPS patients. PBMCs were isolated from the blood using Ficoll-Hypaque (227288; Greiner Bio-One) density gradient centrifugation. Human monocytes were purified from freshly isolated PBMCs using the Dynabeads FlowComp Human CD14 kit (11367D; Thermo Fisher Scientific) according to the manufacturer’s instructions. After isolation, PBMCs and monocytes were stored in liquid nitrogen for later usage. Upon thawing, PBMCs and monocytes were washed in cold DPBS (CA PBS-1A; Capricorn Scientific) and seeded at a density of 8 × 10^4^ cells per well (PBMCs) or 2 × 10^5^ cells per well (monocytes) in tissue culture–treated 96-well plates in RPMI 1640 medium with stable glutamine (CA RPMI-STA; Capricorn Scientific) supplemented with 10% endotoxin-free heat-inactivated fetal bovine serum (S-FBS-SA-015; Bodinco), 100 U ml^−1^ penicillin, and 100 mg ml^−1^ streptomycin (LO DE17-602E; Lonza). Cells were maintained at 37°C in a humidified atmosphere containing 5% CO_2_. All reported patients and healthy donors provided written informed consent for participation in the study, in accordance with ICH/GCP guidelines. The research protocol was approved by the Local Ethics Committee of Ghent University Hospital and the Ethical Committee of the Gaslini Institute in Genoa, Italy. Approval for the use of “blood products unsuitable for transfusion” was obtained with the Red Cross (G20221107A). Treating physicians provided information regarding the CAPS genotype.

### BMDM and iBMDM culture

Bone marrow cells derived from C57BL/6J mice were immortalized as reported (Wang et al, 2006). Primary *Nlrp3*^*A350V/+*^*CreT*^*+*^ BMDMs were obtained as described (Vande Walle et al, 2019). In brief, the CAPS mouse model *Nlrp3*^*A350VneoR*^ (Brydges et al, 2009) was bred to the tamoxifen-inducible Cre line *R26-CreERT2* (Ventura et al, 2007) to generate *Nlrp3*^*A350neoR/+*^*R26-Cre*^*ERT2*^*Tg*^*+*^ (here referred to as *Nlrp3*^*A350V/+*^*CreT*+). Expression of mutant NLRP3 in *Nlrp3*^*A350V/+*^*CreT*+ mice was induced in 6- to 12-wk-old mice by administering 5 mg tamoxifen (T5648; Sigma-Aldrich, dissolved in 0.5:9.5 ethanol/corn oil [C-8267; Sigma-Aldrich] at 50 mg ml^−1^) per mouse per day for two consecutive days. On day 3 post-tamoxifen, bone marrow was isolated for ex vivo analysis. Primary bone marrow cells from wild-type C57BL/6J, *Nlrp3*^*A350V/+*^*CreT−*, or *Nlrp3*^*A350V/+*^*CreT+* mice and immortalized bone marrow cells were differentiated into macrophages in IMDM (LO BE12-722F; Lonza) supplemented with 10% FBS, 30% L929-conditioned medium, 1% nonessential amino acids (LO BE13-114E; Lonza), 100 U ml^−1^ penicillin, and 100 mg ml^−1^ streptomycin at 37°C in a humidified atmosphere containing 5% CO_2_. After 6 d of differentiation, primary BMDMs and iBMDMs were collected and seeded at varying densities, dependent on the analysis being performed, in IMDM containing 10% FBS, 1% nonessential amino acids, along with antibiotics. For immunoblotting, cells were seeded at 4.25 × 10^5^ cells per well in tissue culture–treated 24-well plates. For cytokine analysis, cells were seeded at a density of 1 × 10^5^ cells per well in 96-well plates and used for experiments on the following day. Animal experiments were conducted with permission of the Ethical Committee on Laboratory Animal Welfare of Ghent University and performed in accordance with the guidelines and regulations associated with protocol numbers EC2017-090 and ECD20-90.

### Reagents and stimulation

For analysis of inflammasome activation in wild-type BMDMs and iBMDMs, cells were either left untreated or primed with 100 ng ml^−1^ ultrapure LPS from *Salmonella minnesota* (tlrl-smlps; Invivogen) for 3 h before incubation with vehicle (DMSO) or the indicated concentrations of NIC-11, NIC-12, or CRID3 (5.38120, Sigma-Aldrich) for 30 min. Subsequently, cells were subjected to stimulation with 10 μM nigericin (N-7143; Sigma-Aldrich) or 5 mM ATP (10519987001; Roche) for 45 min or exposed to 500 ng ml^−1^ silica (TLRL-SIO-2; Invivogen) or 1 μg ml^−1^ TcdA from *Clostridium difficile* (BML-G140-0050; Enzo Life Sciences) for 6 h or treated with NdTox, which consists of 500 ng ml^−1^ anthrax protective antigen (PA, NR-140; BEI Resources) and 100 ng ml^−1^ LFn-Needle (TLRL-NDL; Invivogen) for 5 h. In other experiments, cells were treated with transfection agent alone (mock) or transfected with 2 μg ml^−1^ herring sperm dsDNA (15634017; Invitrogen) using Lipofectamine 2000 reagent (11668019; Invitrogen) for 16 h. Primary BMDMs from tamoxifen-treated *Nlrp3*^*A350V/+*^*CreT*− and *Nlrp3*^*A350/V+*^*CreT+* mice were treated with either vehicle (DMSO) or the indicated concentrations of NIC-11, NIC-12, or CRID3 for 30 min, before stimulation with 100 ng ml^−1^ LPS for 3 h. In some experiments, cells were in addition stimulated with 10 μM nigericin for 30 min. To assess cytotoxicity of the compounds, primary BMDMs were treated with either vehicle (e.g., 0.1% DMSO) or 10 μM NIC-11, NIC-12, or CRID3 for 24 h. To assess the effect of the compounds on TLR activity, primary BMDMs were treated with either vehicle (0.01% DMSO) or 1 μM NIC-11, NIC-12, or CRID3 for 30 min, followed by stimulation with 100 ng ml^−1^ LPS, 100 ng ml^−1^ Pam3CSK4 (TLRL-PMS; Invivogen), or 2 μg ml^−1^ Resiquimod (R848, TLRL-R848; Invivogen) for 6 h. For NLRP3 inflammasome activation in human cells, PBMCs or monocytes were treated with vehicle (DMSO) or the indicated concentrations of NIC-11, NIC-12, or CRID3 for 30 min, followed by stimulation with 100 ng ml^−1^ LPS for 6 h. To investigate NLRP3 inflammasome activation downstream of the cGAS/STING pathway, monocytes were either left untreated or primed with 100 ng ml^−1^ Pam3CSK4 for 2 h. Subsequently, monocytes were incubated with either vehicle (e.g., 0.01% DMSO) or 1 μM NIC-11, NIC-12, or CRID3 for 30 min, followed by stimulation with 50 μg ml^−1^ 2'3'-cGAMP (S7904; Selleckchem) for 16 h. To analyze human pyrin or NLRC4 inflammasome activation, Pam3CSK4-primed monocytes were treated with either vehicle (e.g., 0.01% DMSO) or 1 μM NIC-11, NIC-12, or CRID3 for 30 min before stimulation with, respectively, 1 μg ml^−1^ TcdA or NdTox for 5 h.

### Cytokine analysis and LDH measurement

Cytokine levels in culture medium and IL-6 levels in mouse serum were determined by magnetic bead–based multiplex assay using Luminex technology (Bio-Rad). Serum IL-1β levels were determined using IL-1β ELISA (MLB00C; R&D Systems). LDH activity in the culture medium was assayed using CytoTox 96 Non-Radioactive Cytotoxicity Assay (G1780; Promega).

### Immunoblotting

Cell lysates and culture supernatants were incubated with cell lysis buffer (20 mM Tris–HCl [pH 7.4], 200 mM NaCl, 1% Nonidet P-40) and were denatured in lithium dodecyl sulfate sample buffer (M00676; GenScript) and boiled at 95°C for 10 min. SDS-PAGE–separated proteins were transferred to PVDF membranes and immunoblotted with primary antibodies against caspase-1 (AG-20B-0042-C100; Adipogen), IL-1β (GTX74034; Genetex), GSDMD (ab219800; Abcam), and β-actin (SC-47778HRP; Santa Cruz Biotechnology). Peroxidase-AffiniPure Goat Anti-Mouse (115-035-146; Jackson Immunoresearch Laboratories) or Anti-Rabbit (111-035-144; Jackson Immunoresearch Laboratories) secondary antibodies were used to detect proteins by enhanced chemiluminescence (34580; Thermo Fisher Scientific).

### NLRP3 NanoBRET target engagement assay

The NLRP3 NanoBRET target engagement assay was acquired from Promega through the Elite Access program and used following the manufacturer’s guidelines. Briefly, HEK293 cells were transiently transfected with a plasmid encoding a full-length NLRP3-Nanoluc fusion protein using FuGENE HD (E2311; Promega). 24 h after transfection, cells were collected by trypsinization, resuspended in Opti-MEM without phenol red (11058021; Thermo Fisher Scientific), and reseeded at a density of 2 × 10^5^ cells ml^−1^ in white, nonbinding-surface 96-well plates (3990; Corning). Subsequently, cells were treated with serially diluted NIC-11, NIC-12, CRID3, or the caspase-1 inhibitor VX765 in the absence or presence of 0.16 μM NanoBRET NLRP3 tracer (Promega). After 2 h of equilibration, NanoBRET substrate/inhibitor solution (Promega) was added according to the manufacturer’s instructions and BRET was recorded on a SpectraMax i3x plate reader (Molecular Devices) by measuring donor emission (450 nm) and acceptor emission (610 nm). NanoBRET ratios were generated by dividing the acceptor emission value by the donor emission value. For background correction, the BRET ratio in the absence of tracer was subtracted from the BRET ratio of each sample. The following equation is used to determine the NanoBRET ratio: ([Acceptor_sample_/Donor_sample_] – [Acceptor_no tracer control_/Donor_no tracer control_]) × 1,000. IC_50_ values were determined through nonlinear regression curve fitting.

### Structural modeling

The crystal structure of the NLRP3 NACHT domain bound to NP3-562 (PDB ID: 8RI2) was retrieved from the Protein Data Bank (PDB) and used as a template to build a model of the NIC-12–bound structure. To rebuild the ligand, the scaffold of NP3-562 was edited in Molecular Operating Environment (MOE), replacing the isopropanol moiety by an isopropyl group and the N-methyl by N-methylcyclopropyl. Docking of NIC-12 to the NLRP3 NACHT domain was performed using PyMOL 2.0’s built-in docking module. The MMFF94x force field was used to minimize the three-dimensional structure of NIC-12, while keeping the protein, water, and ADP ligand fixed. After minimization, strain energy analysis indicated that the N-methylcyclopropyl group prefers to adopt an axial orientation. The group was rebuilt using an axial orientation and minimized using appropriate force fields and optimization algorithms available in PyMOL. The final complexes with NIC-12 and CRID3 were visually inspected and rendered using PyMOL. Interactions between NIC-12 and the NLRP3 NACHT domain were mapped using MOE.

### In vivo LPS challenge

6- to 12-wk-old mice were intraperitoneally injected with either vehicle solution or 50 mg kg^−1^ NIC-12 or CRID3, 30 min before receiving an intraperitoneal challenge with either PBS or 10 mg kg^−1^ LPS (*Escherichia coli*, serotype 0111:B4, L-2630; Sigma-Aldrich). 5% DMSO in PBS was used as a vehicle control for NIC-12, whereas PBS was used as a vehicle control for CRID3. Mice were euthanized 3 h after LPS challenge for blood collection. Animal experiments were conducted with permission of the Ethical Committee on Laboratory Animal Welfare of Ghent University and performed in accordance with the guidelines and regulations associated with protocol number ECD22-06.

### Carbonic anhydrase I and II enzymatic assay

5 μg ml^−1^ recombinant carbonic anhydrase I (CA I, C4396; Sigma-Aldrich) and 0.5 μg ml^−1^ CA II (C6165, Sigma-Aldrich) were pretreated with 100 μM CRID3, NIC-11, or NIC-12 for 15 min in assay buffer (12.5 mM Tris, 75 mM NaCl, pH 7.5) before incubation with 1 mM 4-nitrophenyl acetate substrate (p-NPA, N8130; Sigma-Aldrich). Esterase activity was assessed by monitoring the formation of 4-nitrophenol at 405 nm every 90 s at 27°C for 1 h.

### Statistics

All data were analyzed using Prism 10.0.2 software. Data are shown as mean ± SEM of three or more independent biological replicates. For cell death kinetics, cytokine analysis in supernatants, and the CA activity assay, parametric one-way ANOVA with Bonferroni’s multiple-comparison test was used after confirming normal distribution using the Shapiro–Wilk test. For cell death kinetics and cytokine analysis in supernatants from different mouse genotypes, we used two-way ANOVA with Bonferroni’s multiple-comparison test. Serum cytokine analysis was subjected to the Kolmogorov–Smirnov test to assess normality, followed by one-way ANOVA with Bonferroni’s multiple-comparison test. ****P ≤ 0.0001; ***P ≤ 0.001; **P ≤ 0.01; *P ≤ 0.05; ns, nonsignificant.

### Curve fitting

All dose–response curves represent n = 3 or more, with error bars indicating SEM. In studies involving mouse primary BMDMs and human primary monocytes, compounds were tested in a threefold dilution series starting at 10 μM. In human PBMCs, compounds were assessed in a fivefold dilution series starting at 50 μM. To obtain the percentage of inhibition (% inhibition), values measured were normalized to a no-inhibition control (MAX; DMSO + stimulus) and a full-inhibition control (MIN; DMSO + culture medium). To calculate the % inhibition at a specific inhibitor concentration (denoted as x), the following equation was used: % inhibition = 100 − ([x–MIN]/[MAX - MIN]) × 100. IC_50_ and pIC_50_ values were determined through nonlinear regression curve fitting.

## Supplementary Material

Reviewer comments

## Data Availability

All data to understand and assess the conclusions of this research are available in the main text and supplementary materials.
