# Supplementary material for: Novel chemotype NLRP3 inhibitors that target the CRID3-binding pocket with high potency
Source: Life Sci Alliance. 2024 Mar 22;7(6):e202402644. doi: 10.26508/lsa.202402644 (PMC10961714; doi:10.26508/lsa.202402644)
Supplement: Supplementary file 1 [file LSA-2024-02644_SdataF1.pdf]

Full immunoblot of Fig 1K

CASP1

α-Casp-1 (AG-20B-0042-C100, AdipoGen); 1:1000 in milk buffer (PBS, 0.05% TWEEN 20, 3% milk).

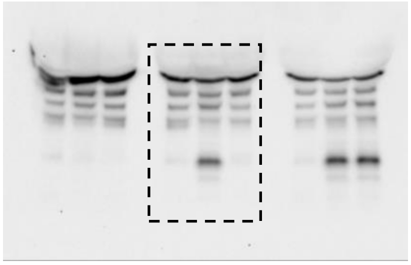

NIC-11

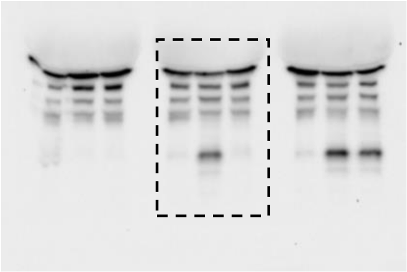

NIC-12

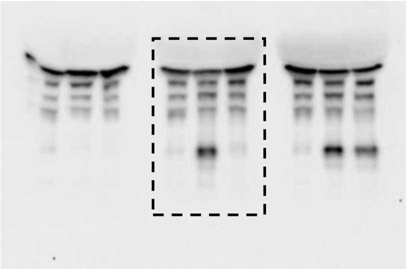

CRID3

IL-1β

α-IL-1β (GTX74034, GeneTex); 1:3000 in milk buffer (PBS, 0.05% TWEEN 20, 3% milk).

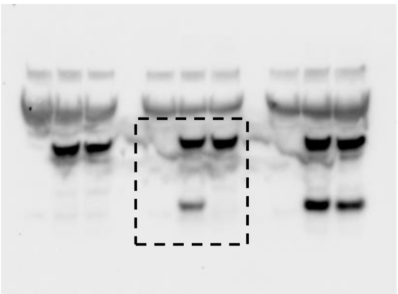

NIC-11

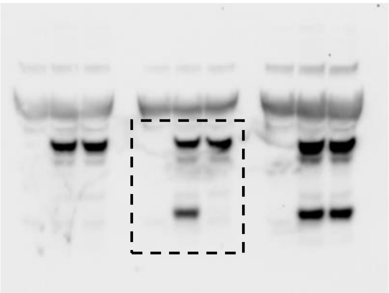

NIC-12

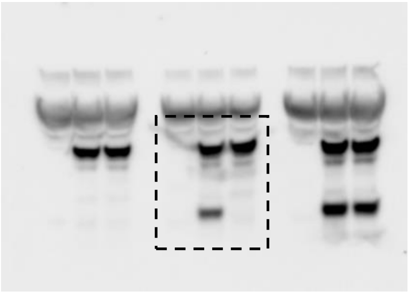

CRID3

GSDMD

α-GSDMD (ab219800, Abcam); 1:2000 in milk buffer (PBS, 0.05% TWEEN 20, 3% milk).

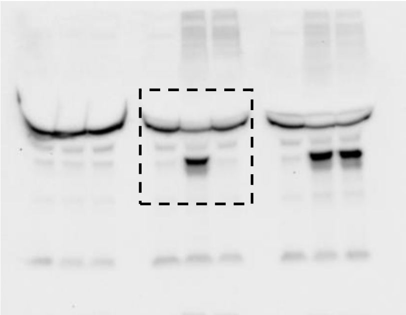

NIC-11

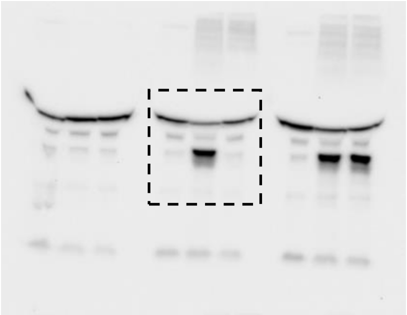

NIC-12

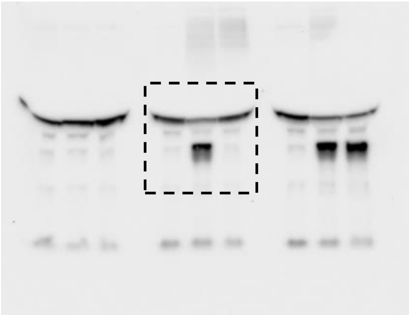

CRID3

β-actin

α-β-actin (SC-47778HRP, Santa Cruz Biotechnology); 1:2000 in milk buffer (PBS, 0.05% TWEEN 20, 3% milk).

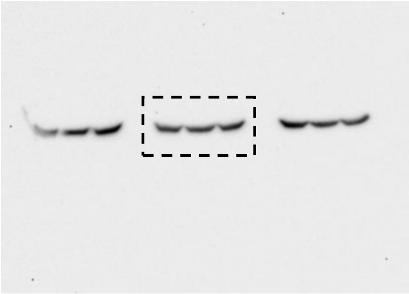

NIC-11

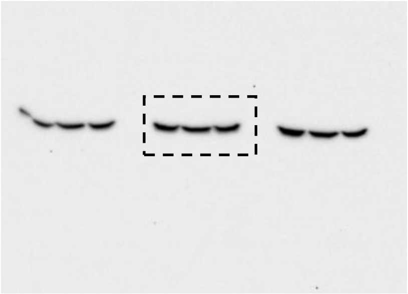

NIC-12

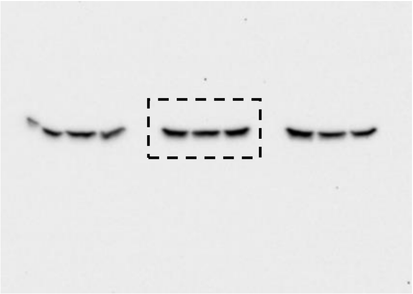

CRID3
