# Supplementary material for: Novel chemotype NLRP3 inhibitors that target the CRID3-binding pocket with high potency
Source: Life Sci Alliance. 2024 Mar 22;7(6):e202402644. doi: 10.26508/lsa.202402644 (PMC10961714; doi:10.26508/lsa.202402644)
Supplement: Supplementary file 2 [file LSA-2024-02644_Supplemental_Data_1.docx]

**Supplementary Methods - Chemical synthesis**

**4H-thieno [3,2-b]pyrrole-5-carbohydrazide (2)**

In a flame-dry round bottom flask, methyl 4H-thieno[3,2-b]pyrrole-5-carboxylate 1a (5 g, 1.0 mmol) was dissolved in an anhydrous EtOH (200 mL). The hydrazine monohydrate (7.7 g, 5.0 mmol) was added at room temperature. The reaction mixture was heated under reflux (90 °C) for 16 h. The reaction mixture was cooled to 0 ^°^C and the resulting precipitate was filtered and washed with cold EtOH (50 mL). The resulting solid was dried in vacuo to obtain the desired product (5.7 g, 87%), a white solid.

R_f_ 0.10 (30% EtOAc/n-hexane); IR (CHCl_3_) ν_max_: 3299 (s), 3238 (br), 1620 (s), 1549 (s), 1510 (s), 1401 (s), 1366 (s), 1319 (s), 1233 (s), 1149 (s), 814 (s) cm^-1^; ^1^H NMR (400 MHz, DMSO-*d*_6_) δ: 11.72 (br. s., 1H), 9.49 (br. s., 1H), 7.36 (d, *J* = 5.3 Hz, 1H), 7.04 (d, *J* = 1.6 Hz, 1H), 6.94 (d, *J* = 5.2 Hz, 1H), 4.39 (br. s., 2H) ppm; ^13^C{^1^H} NMR (100 MHz, DMSO-*d*_6_) δ: 161.3, 140.4, 129.7, 127.0, 122.8, 111.9, 101.5 ppm; HRMS (ESI-TOF): m/z calculated for C_7_H_8_N_3_OS^+^ [M+H]^+^ 182.039, found 182.038.

**5-isopropylthieno[2',3':4,5]pyrrolo[1,2-d][1,2,4]triazin-8(7H)-one (3)**

To a stirred solution of hydrazide **2** (4 g, 1.0 mmol) in DMF (50 mL) was added trimethyl orthopropionate **2a** (3.9 g, 1.2 mmol) at room temperature. The reaction mixture was heated at 140 °C for 24 h. The reaction mixture was then cooled to room temperature and the solvent was evaporated in a vacuum. The crude product was purified using column chromatography to obtain the desired product in **3** (4.2 g, 84%) as a white solid.

R_f_ 0.3 (30% EtOAc/n-hexane); IR (CHCl_3_) ν_max_: 3122 (br), 2981 (br), 1604 (s), 1568 (s), 1147 (s), 1350 (s), 1188 (s), 1148 (s), 806 (s), 719 (s) cm^-1^; ^1^H NMR (400 MHz, DMSO-*d*_6_) δ: 12.37 (s, 1H), 7.47 (d, *J* = 5.2 Hz, 1H), 7.11 (s, 1H), 7.03 (d, *J* = 5.2 Hz, 1H), 3.25 (spt, *J* = 7.0 Hz, 1H), 1.36 (d, *J* = 7.0 Hz, 6H) ppm; ^13^C{^1^H} NMR (100 MHz, DMSO-*d*_6_) δ: 169.2, 159.1, 142.2, 128.4, 123.8, 120.1, 111.7, 103.8, 25.6, 19.8 ppm; HRMS (ESI-TOF): m/z calculated for C_11_H_12_N_3_OS^+^ [M+H]^+^ 234.070, found 234.070.

**Ethyl 2-(5-isopropyl-8-oxothieno[2',3':4,5]pyrrolo[1,2-d][1,2,4]triazin-7(8H)-yl)acetate (4)**

In a flame-dry round bottom flask ester **3** (3 g, 1.0 mmol), was dissolved in dry ACN. The K_2_CO_3_ (3.51g, 2.0 mmol) and ethyl 2-iodoacetate **3a** were added at room temperature. The reaction mixture was heated at 60 °C for 12 h. The reaction mixture was diluted using water and extracted with EtOAc (3 times). The combined organic layer was dried over the Na_2_SO_3_ and concentrated in a vacuum. The residue was purified by column chromatography to yield the product **4** (3.7 g, 91%) as white solid.

R_f_ 0.6 (30% EtOAc/n-hexane); IR (CHCl_3_) ν_max_: 2984 (w), 1749 (s), 1600 (s), 1561 (s), 1438 (s), 1201 (s), 1024 (s), 719 (s) cm^-1^; ^1^H NMR (400 MHz, DMSO-*d*_6_) δ: 7.54 (d, *J* = 5.3 Hz, 1H), 7.31 (d, *J* = 5.3 Hz, 1H), 7.24 (s, 1H), 5.45 (s, 2H), 4.14 (q, *J* = 7.1 Hz, 2H), 3.25 (spt, *J* = 6.8 Hz, 1H), 1.34 (d, *J* = 7.0 Hz, 6H), 1.19 (t, *J* = 7.1 Hz, 3H) ppm; ^13^C{^1^H} NMR (100 MHz, DMSO-*d*_6_) δ: 168.9, 168.6, 158.5, 145.1, 128.8, 122.5, 121.3, 111.5, 105.8, 61.0, 48.9, 25.5, 19.7, 14.1 ppm; HRMS (ESI-TOF): m/z calculated for C_15_H_18_N_3_O_3_S^+^ [M+H]^+^ 320.107 found 320.107.

**Ethyl 2-(2-chloro-5-isopropyl-8-oxothieno[2',3':4,5]pyrrolo[1,2-d][1,2,4]triazin-7(8H)-yl)acetate (5):**

In a flame-dry round bottom flask, ester **4** (3 g, 1.0 mmol) was dissolved in dry THF. Recrystallized NCS (6.8 g, 3.0 mmol) was added at room temperature and the reaction mixture was heated at 55 °C for 3 h. Another portion of NCS (2.2 g, 1.0 mmol) was added and the mixture was heated for 3 h. The reaction mixture was quenched with 10% of NaHCO_3_ and extracted using EtOAc (3 times). The combined organic layer was washed with brine and dried over Na_2_SO_4_. The organic layer was concentrated and the residue was purified using column chromatography. Compound **5** (2.8 g, 85%) was obtained as a white solid.

R_f_ 0.5 (20% EtOAc/n-hexane); IR (CHCl_3_) ν_max_: 2980 (s), 1751 (s), 1602 (s), 1562 (s), 1437 (s), 1212 (s) , 1172 (s), 1051 (s) 951 (s) cm^-1^; 1H NMR (400 MHz, CDCl_3_) δ: 7.01 (s, 1H), 6.84 (s, 1H), 5.35 (s, 2H), 4.22 (d, *J* = 7.2 Hz, 2H), 3.24 (spt, *J* = 6.9 Hz, 1 H), 1.42 (d, *J* = 7.0 Hz, 6 H), 1.27 (t, *J* = 7.1 Hz, 3 H) ppm; ^13^C{^1^H} NMR (100 MHz, CDCl_3_) δ: 168.9, 168.6, 158.5, 145.1, 128.8, 122.5, 121.3, 111.5, 105.8, 61.0, 48.9, 25.5, 19.7, 14.1 ppm; HRMS (ESI-TOF): m/z calculated for C_15_H_17_ClN_3_O_3_S^+^ [M+H]^+^ 354.068, found 354.067.

**2-(2-chloro-5-isopropyl-8-oxothieno[2',3':4,5]pyrrolo[1,2-d][1,2,4]triazin-7(8H)-yl)acetic acid (6)**

The ester **5** (1.0 g 1.0 mmol) dissolved in 20 mL of THF. The reaction mixture was treated with 1M of LiOH (10 mL) and stirred at room temperature for 12 h. Next, the solution was acidified using 2M HCl and diluted with water. The water layer was extracted with DCM and dried over Na_2_SO_4_. The organic layer was concentrated, and the desired compound was obtained as a white solid (0.73 g, 80%) and used for the next step without purification.

**Tert-butyl (R)-(1-(cyclopropylmethyl)piperidin-3-yl)carbamate (8)**

In a round bottom flask tert-butyl (*R*)-piperidin-3-ylcarbamate **7** (0.2 g 1.0 mmol) was dissolved in a dry ACN. Then DIPEA (0.258 g, 2.0 mmol) and (bromomethyl)cyclopropane (0.2 g, 1.5 mmol) were added at room temperature. The reaction mixture was stirred at 50 °C for 12 h. The water was added to the reaction mixture, extracted with EtOAc (3 times) and dried over Na_2_SO_4_. The combined organic layer was concentrated, and the residue was purified using column chromatography to yield product **8** (0.22 g, 90%) as a white solid.

R_f_ 0.3 (100% EtOAc); IR (CHCl_3_) ν_max_: 3355 (s) , 2936 (s), 1678 (s), 1522 (s), 1311 (s), 1244 (s), 1160 (s), 1110 (s), 980 (s) cm^-1^; ^1^H NMR (400 MHz, CDCl_3_) δ: 5.01 (br. s., 1H), 3.75 (m, 1H), 2.48 (m, 4H), 2.21 (d, *J* = 6.5 Hz, 2H) 1.76 - 1.67 (m, 1H), 1.66 - 1.53 (m, 2H), 1.45 (s, 9H), 0.89 - 0.76 (m, 1H), 0.53 - 0.46 (m, 2H), 0.09 - 0.05 (m, 2H) ppm; ^13^C{^1^H} NMR (100 MHz, CDCl_3_) δ: 155.2, 78.9, 63.8, 58.6, 53.7, 46.3, 28.4, 8.2, 3.9, 3.9 ppm; HRMS (ESI-TOF): m/z calculated for C_14_H_27_N_2_O_2_^+^ [M+H]^+^ 255.207, found 255.207.

**(R)-1-(cyclopropylmethyl)piperidin-3-amine (9)**

To tert-butyl (*R*)-(1-(cyclopropylmethyl)piperidin-3-yl)carbamate (0.2 g, 1.0 mmol) dissolved in 1 ml of methanol was added 1.5 M HCl/MeOH (2 mL) at 15 °C and stirred for 1 h. The reaction mixture was concentrated in a vacuum to obtain compound **9** as a white solid (0.12 g, 99 %), which was used in the next step without purification.

IR (CHCl_3_) ν_max_: 3477 (br), 2972 (w), 1733 (w), 1602 (s), 1567 (s), 1433 (w) 1388 (s), 949 (s) cm^-1^; ^1^H NMR (400 MHz, MeOD_4_) δ: 3.93 (d, *J* = 10.1 Hz, 1H), 3.82 - 3.62 (m, 2H), 3.28 - 3.00 (m, 4H), 2.24 (d, *J* = 10.9 Hz, 1H), 2.19 - 2.08 (m, 1H), 2.03 (d, *J* = 12.2 Hz, 1H ), 1.75 (m, 1H), 1.30 - 1.12 (m, 1H), 0.79 (d, *J* = 7.7 Hz, 2H), 0.57 - 0.43 (m, 2H) ppm; ^13^C{^1^H} NMR (100 MHz, MeOD_4_) δ: 63.5, 53.5, 52.9, 46.8, 27.6, 22.2, 6.5, 5.4, 5.2 ppm; HRMS (ESI-TOF): m/z calculated for C_9_H_19_N_2_^+^ [M+H]^+^ 155.155 found 155.154.

**Target Compound NIC-11**: **2-(2-chloro-5-(2-hydroxypropan-2-yl)-8-oxothieno [2',3':4,5]pyrrolo[1,2-d][1,2,4]triazin-7(8H)-yl)-N-(pyrimidin-4-yl)acetamide (NIC-11)**

NIC-11 was prepared from compound **1** as described in **Supplementary Figure 1b**. Following a synthesis path paralleling that described above for NIC-12, acid **14** and 2-aminopyrimidine **15** were dissolved in dry DMF (5 mL) in a round bottom flask under the argon. DIPEA and HATU were added into the reaction mixture. The solution was stirred at 20 °C for 1 h, followed by evaporation of DMF in a vacuum. The residue was dissolved in water and extracted using EtOAc. The obtained product was purified using preparative TLC using 30% MeOH/ EtOAc to obtain **NIC-11** (13 mg, 16%) as a yellow solid.

R_f_ 0.5 (10% MeOH/ EtOAc); IR (CHCl_3_) ν_max_: 3312 (br), 2985 (w), 1703 (s), 1633 (s), 1580 (s), 1531 (s), 1420 (s), 1391 (s), 1310 (s), 1185 (s), 1088 (w), 999 (w) cm^-1^; ^1^H NMR (400 MHz, DMSO-*d*_6_) δ: 11.27 (br. s., 1 H), 8.92 (s, 1 H), 8.67 (d, *J* = 5.8 Hz, 1 H), 7.99 (d, *J* = 5.7 Hz, 1 H), 7.92 (s, 1 H), 7.57 (s, 1 H), 6.14 (s, 1 H), 4.90 (s, 2 H), 1.59 (s, 6 H) ppm; ^13^C{^1^H} NMR (100 MHz, DMSO-*d*_6_) δ: 167.7, 158.5, 158.4, 157.3, 153.2, 140.7, 132.7, 129.5, 126.9, 126.6, 118.9, 109.9, 105.0, 71.2, 52.7, 28.1 ppm; HRMS (ESI-TOF): m/z calculated for C_17_H_16_ClN_6_O_3_S^+^ [M+H]^+^ 419.0 found 419.0.

**Target Compound NIC-12**: 2-(4-chloro-9-oxo-12-propan-2-yl-5-thia-1,10,11-triazatricyclo [6.4.0.02,6] dodeca-2(6),3,7,11-tetraen-10-yl)-N-[(3R)-1-(cyclopropylmethyl)piperidin-3-yl]acetamide.

NIC-12 was prepared according to **Supplementary Figure 1a**. In a round bottom flask acid **6** (50 mg 1.0 mmol) and amine **9** (26 mg, 1.0 mmol) were dissolved in dry DMF (5 mL) under the argon. DIPEA (90 mg, 3.0 mmol) and HATU (96 mg, 1.5 mmol) were added into the reaction mixture. The solution was stirred at room temperature for 3 h, followed by evaporation of DMF in a vacuum. The residue was dissolved in water and extracted using EtOAc (3 times). The obtained product was purified using preparative TLC using 30% MeOH/ EtOAc to obtain **NIC-12** (21 mg, 30%) as a white solid.

R_f_ 0.3 (30% MeOH/ EtOAc); IR (CHCl_3_) ν_max_: 3297 (br), 2920 (s), 1661 (s), 1603 (s), 1565 (s), 1439 (s), 1261 (s), 1095 (w), 803 (w) cm^-1^; ^1^H NMR (400 MHz, MeOD_4_) δ: 7.16 (s, 1H), 7.13 (s, 1H), 5.28 (s, 2H), 3.96 – 3.86 (m, 1H), 3.29 – 3.22 (m, 2H), 3.02 – 2.87 (m, 1H), 2.87 – 2.72 (m, 1H), 2.25 (d, *J* = 6.6 Hz, 2H), 2.19 – 2.12 (m, 1H), 2.09 – 2.00 (m, 1H), 1.82 (dd, *J* = 3.7, 12.1 Hz, 1H), 1.75 – 1.68 (m, 1H), 1.64 – 1.56 (m, 2H), 1.43 (d, *J* = 7.0 Hz, 7H), 0.88 (dd, *J* = 1.9, 4.5 Hz, 1H), 0.85 (d, *J* = 6.6 Hz, 1H), 0.51 (dd, *J* = 1.3, 8.0 Hz, 2H), 0.13 – 0.08 (m, 2H) ppm; ^13^C{^1^H} NMR (100 MHz, MeOD_4_) δ: 171.3, 169.3, 160.7, 143.6, 133.1, 122.5, 122.4, 112.4, 107.3, 64.8, 59.1, 54.5, 51.2, 47.6, 31.0, 31.0, 27.6, 20.4 (×2), 8.9, 4.7, 4.6 ppm; HRMS (ESI-TOF): m/z calculated for C_22_H_29_ClN_5_O_2_S^+^ [M+H]^+^ 462.173, found 462.173.
