# Supplementary material for: Novel chemotype NLRP3 inhibitors that target the CRID3-binding pocket with high potency
Source: Life Sci Alliance. 2024 Mar 22;7(6):e202402644. doi: 10.26508/lsa.202402644 (PMC10961714; doi:10.26508/lsa.202402644)
Supplement: Supplementary file 3 [file LSA-2024-02644_Supplemental_Data_2.docx]

**NMR spectra of NIC-11 and NIC-12**

**2-(2-chloro-5-(2-hydroxypropan-2-yl)-8-oxothieno[2',3':4,5]pyrrolo[1,2-d][1,2,4]triazin-7(8H)-yl)-N-(pyrimidin-4-yl)acetamide (NIC-11)**

**^1^H NMR, 400 MHz, DMSO-*d*_6_ (NIC-11)**


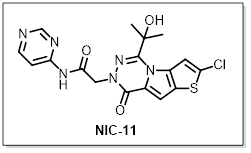


**^13^C NMR, 400 MHz, DMSO-*d*_6_ (NIC-11)**

**(R)-2-(2-chloro-5-isopropyl-8-oxothieno[2',3':4,5]pyrrolo[1,2-d][1,2,4]triazin-7(8H)-yl)-N-(1-(cyclopropylmethyl)piperidin-3-yl)acetamide (NIC-12)**

**^1^H NMR, 400 MHz, CD_3_OD (NIC-12****)**

**^13^C NMR, 400 MHz, CD_3_OD (NIC-12)**
